# RNA Probes for Visualization of Sarcin/ricin Loop Depurination without Background Fluorescence

**DOI:** 10.1002/asia.202201077

**Published:** 2022-11-18

**Authors:** Robin Klimek, Christoph Kaiser, Nina S. Murmann, Nina Kaltenschnee, Teresa Spanò, Josef Wachtveitl, Erin M. Schuman, Alexander Heckel

**Affiliations:** ^1^ Institute of Organic Chemistry and Chemical Biology Goethe-University Frankfurt Max-von-Laue Str. 7–9 60438 Frankfurt Germany; ^2^ Institute of Physical and Theoretical Chemistry Goethe-University Frankfurt Max-von-Laue Str. 7–9 60438 Frankfurt Germany; ^3^ Teresa Spanò Max Planck Institute for Brain Research Max-von-Laue Str. 4 60438 Frankfurt Germany

**Keywords:** Sarcin-ricin-loop, molecular beacon, fluorescence, ribosome, depurination

## Abstract

Protein synthesis via ribosomes is a fundamental process in all known living organisms. However, it can be completely stalled by removing a single nucleobase (depurination) at the sarcin/ricin loop of the ribosomal RNA. In this work, we describe the preparation and optimization process of a fluorescent probe that can be used to visualize depurination. Starting from a fluorescent thiophene nucleobase analog, various RNA probes that fluoresce exclusively in the presence of a depurinated sarcin/ricin‐loop RNA were designed and characterized. The main challenge in this process was to obtain a high fluorescence signal in the hybridized state with an abasic RNA strand, while keeping the background fluorescence low. With our new RNA probes, the fluorescence intensity and lifetime can be used for efficient monitoring of depurinated RNA.

## Graphical abstract

**Depurination of a single nucleobase** at the sarcin ricin loop can knock out the entire ribosome. So far, however, this biochemical reaction could not be satisfactorily visualized. In this work, we demonstrate the design and characterization of a fluorescent probe that can exclusively detect depurinated RNA.

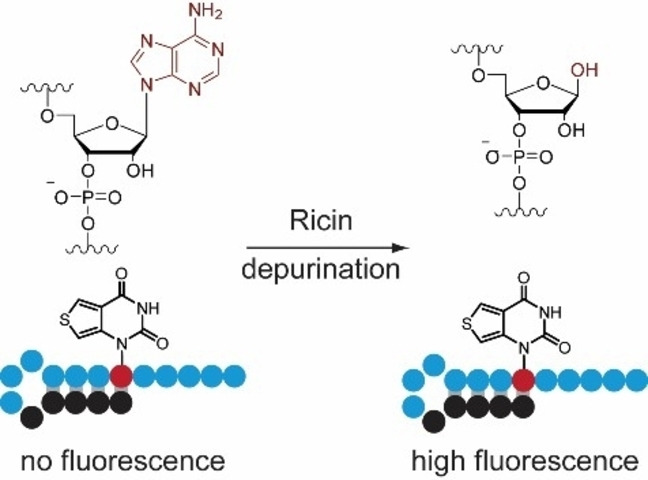

## Introduction

**Figure 1:**
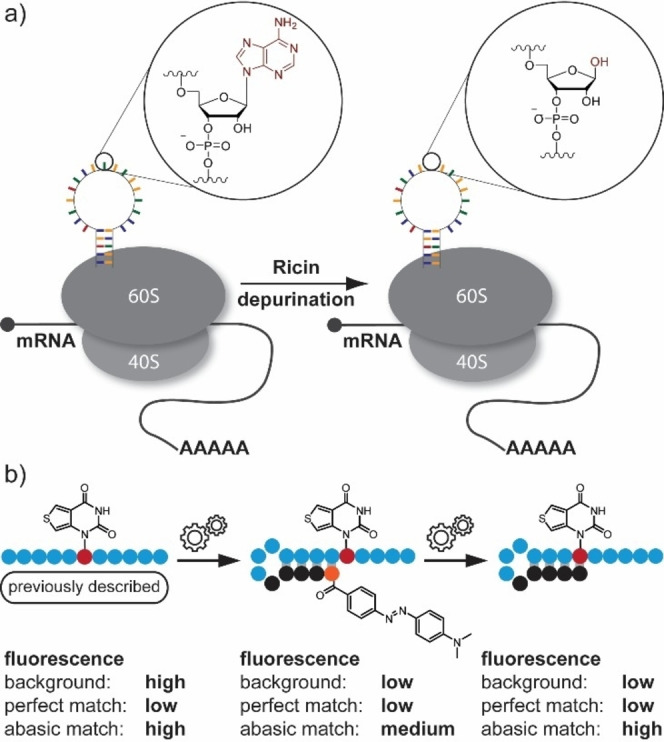
a) General mechanism of sarcin/ricin loop depurination. b) Evolution of RNA probes for abasic site detection. The previously described nucleoside shows a high background fluorescence even without hybridization to corresponding RNA. In this study, we show how to reduce the background fluorescence of unbound probe, while still maintaining a high level of fluorescence for detecting depurination.

The central dogma of molecular biology states that DNA is transcribed to RNA and RNA is translated into proteins. Translation takes place in ribosomes, which are composed of ribosomal RNA and ribosomal proteins, namely the 60S and 40S subunits in eukaryotes and the 50S and 30S subunits in prokaryotes. Even though the structure and composition of ribosomes differ for prokaryotes and eukaryotes, there are also some conserved sequences. These include the so‐called sarcin/ricin loop (SRL), which is located at the surface of the large subunit of the ribosome in the 23S and 28S rRNA in prokaryotes and eukaryotes, respectively.[^1^, ^2^] This sequence forms a stem‐loop structure and interacts with the toxic proteins α‐sarcin and ricin, earning its name.^[3]^ Both proteins are able to inhibit translation very efficiently. However, they differ in the way they interact with the SRL.^[4]^ α‐Sarcin can catalytically cleave the bond between G_4325_ and A_4326_, which are located in the SRL.^[5]^ As a result, stabilization of the catalytic histidine residue by the former phosphodiester bond is no longer possible and translation elongation is inhibited. Ricin, on the other hand, exhibits RNA N‐glycosidase activity at the A‐chain. This enables the selective cleavage of the N‐glycosidic bond of A_4324_ at the SRL (see Figure 1a).[^6^, ^7^] The cleavage results in an abasic site instead of the nucleobase, which means that the stabilization network necessary for the association of elongation factors is disabled. Therefore, the entire protein biosynthesis is inhibited. While the exact mechanism of these catalytic processes has been known for many years, suitable tools to monitor SRL depurination by ricin *in vivo* are still lacking. In general, light is well‐suited as a non‐invasive tool to understand various biological processes.[^8^, ^9^, ^10^] To monitor RNA, particularly fluorescent probes have proven successful in recent years.[^11^, ^12^, ^13^, ^14^, ^15^, ^16^, ^17^] The fluorophores can be attached to the nucleic acid either directly *via* solid phase synthesis,[^18^, ^19^] through chemo‐enzymatic modification,[^20^, ^21^, ^22^, ^23^] reversibly as an aptamer[^24^, ^25^] or chemically, for example using click chemistry[^26^, ^27^] or cycloaddition.^[28]^ Towards detecting depurination with light, Srivatsan and Tor *et al*. (2008) presented a nucleobase analog which fluoresced in a single strand.[^29^, ^30^] When hybridized to a complementary counter strand, it showed strong fluorescence quenching. Hybridized to an abasic site, however, the fluorescence remained very high. Therefore, freely diffusing probe could not be distinguished from the one hybridized to a depurinated site, resulting in a very high background signal. Subsequently, Srivatsan demonstrated a label‐free method for indicating depurination using a fluorescent ligand 2‐amino‐5,6,7‐trimethyl‐1,8‐naphthyridine (ATMND).^[31]^ However, due to the presence of an abasic site, the fluorescence of ATMND was reduced rather than increased using this method. As a result, strong background fluorescence remained a problem, making it difficult to detect depurination in a spatially resolved manner.

In this work, we developed RNA probes based on the fluorescent nucleoside presented by Srivatsan and Tor *et al*.^[29]^ that emit fluorescence exclusively when an abasic site is present (Figure 1b). Our rational RNA architecture allows us for the first time to distinguish freely diffusing, unbound probe from that hybridized to an RNA strand containing an abasic site. We demonstrate the functionality for monitoring depurination on an SRL analog model system.

## Results and Discussion

**Figure 2:**
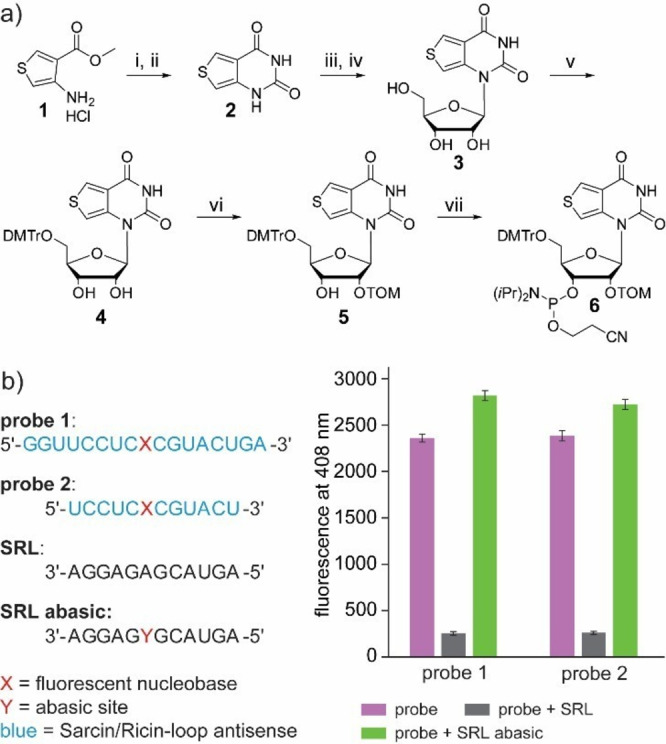
a) Improved synthesis of phosphoramidite **6**. i) KOCN, 86%, ii) NaOMe, 92%, iii) 1‐O‐Acetyl‐2,3,5‐tri‐O‐benzoyl‐*beta*‐D‐ribofuranose, TMSOTf, BSA, iv) MeNH_2_ in MeOH, 42% over two steps, v) DMTr‐Cl, DIPEA, 52%, vi) TOM‐Cl, Bu_2_SnCl_2_, 54%, vii) 2‐cyanoethyl‐N,N‐diisopropyl chlorophosphoramidite, DIPEA, 87%. b) **Probes 1**–**2** and their fluorescence properties when hybridized to complementary **SRL** and **SRL abasic** RNA. c_probe_=10 μM in CSH brain buffer (135 mM NaCl, 5.4 mM KCl, 1 mM MgCl_2_, 5 mM HEPES) at 37 °C, 2 eq. counter strand RNA, λ_ex_=304 nm, v_ges_=100 μL.

To prepare the next generation of depurination‐sensitive RNA probes, it was first necessary to synthesize the phosphoramidite of the fluorescent nucleobase. The synthesis was performed according to the literature[^29^, ^30^] with some improvements in experimental protocols and yields (Figure 2a). Thiophene **1** was reacted with KOCN and subsequently cyclized with NaOMe resulting in the fluorescent nucleobase analogue **2**. In the next step, compound **2** was coupled with 1‐O‐Acetyl‐2,3,5‐tri‐O‐benzoyl‐*beta*‐D‐ribofuranose *via* a Vorbrüggen[^32^, ^33^] mechanism. In contrast to the synthesis known from the literature,[^29^, ^30^] complete purification of the coupling product was omitted because it was difficult to separate from the impurities. Instead, the benzoyl groups were directly cleaved with MeNH_2_ in MeOH. Nucleoside **3** could thus be obtained without complicated purification of the intermediate after column chromatography. Afterwards, nucleoside **3** was selectively protected with a dimethoxytrityl group on the 5’‐OH yielding in compound **4**. Using freshly distilled TOM−Cl, a yield of 54% was achieved in the preparation of compound **5**. The final phosphoramidite **6** was synthesized using 2‐cyanoethyl‐N,N‐diisopropyl chlorophosphor‐amidite and Hünig's base.

To evaluate the fluorescence properties of the synthesized nucleoside in an RNA strand, the oligonucleotides **probe 1** and **probe 2** were prepared (see Figure 2b, left). Both strands are complementary to the SRL and differ only in length. Their sequence was identical to two probes previously described by Srivatsan *et al.*.^[30]^ Furthermore, two RNA strands were synthesized that have the same sequence as the SRL. One of the two strands features an abasic site (a sugar/phosphate backbone with a hydrogen instead of a nucleobase) to function as an analog to the ricin‐depurinated SRL. To mimic the abasic site produced by ricin, we used the commercially available dSpacer phosphoramidite (*GLEN Research*), which provides a stable tetrahydrofuran backbone without nucleobase in the RNA strand (see Supporting Information). The results of the fluorescence studies of **probe 1** and **probe 2** are summarized in Figure 2b (right).

Compared to the fluorescence of the free probes in solution (burgundy), the intensity drops significantly when the probes are matched with a complementary counter strand (grey). However, if the counter strand contains an abasic site opposite the fluorescent nucleoside, the signal intensity remains at about the same level as the free probes (green). This result shows that the synthesized **probe 1** and **probe 2** are suitable for indicating depurination giving a fluorescent signal in our model system, which is in agreement to the measurements Srivatsan *et al*. performed before.^[30]^ However, the clear disadvantage is the similar signal intensity of the free samples without counter strand compared to hybridization with **SRL abasic**. Therefore, the possible applications are severely limited in cells where there is inevitably an excess of the probe.

**Figure 3:**
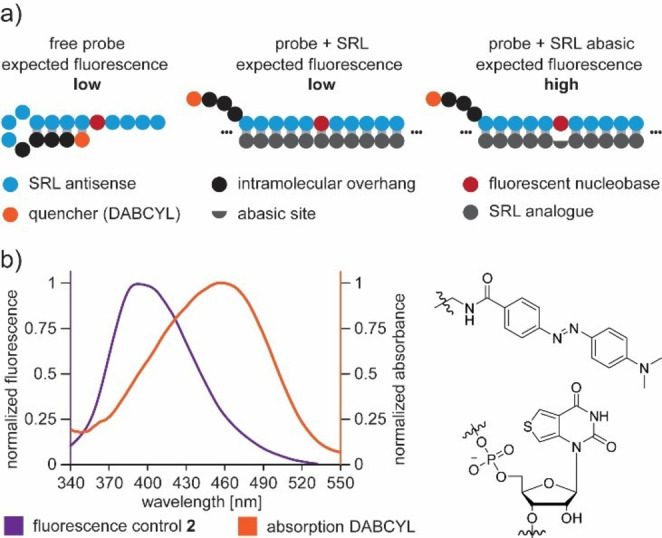
a) Design concept of the new quencher probes with intramolecular RNA overhang. b) Chemical structures (right) and relevant spectra of fluorophore and quencher used for the detection (left).

**Figure 4:**
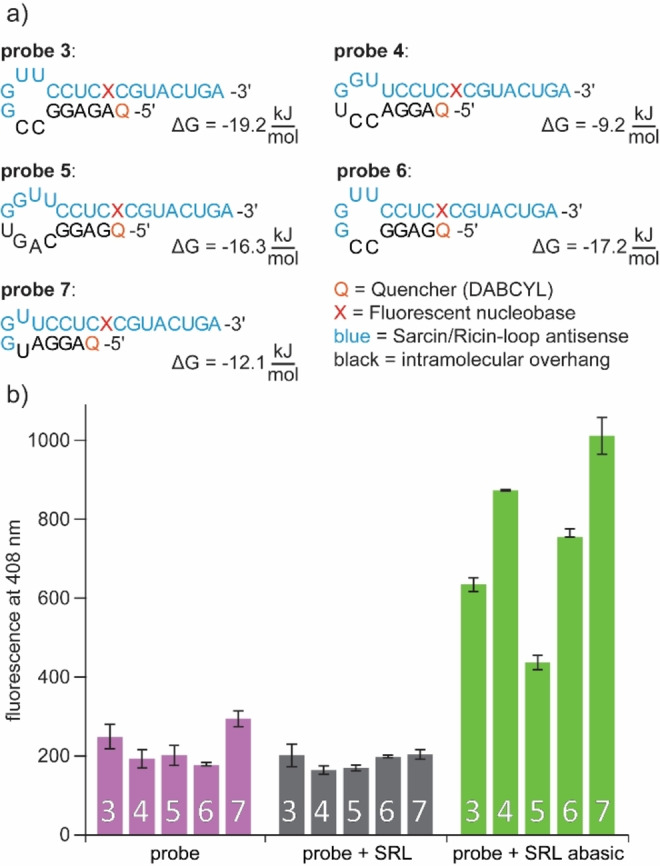
a) Predicted secondary structures and corresponding energies of quencher **probes 3**–**7**. For energy calculation, the fluorescent nucleoside was approximated as uridine. b) Fluorescence intensities at 408 nm of the synthesized probes. c_probe_=10 μM in CSH brain buffer (135 mM NaCl, 5.4 mM KCl, 1 mM MgCl_2_, 5 mM HEPES) at 37 °C, 2 eq counter strand RNA, λ_ex_=304 nm, v_ges_=100 μL.

To overcome this problem, the probes needed to be optimized. For this purpose, we designed new probes, which were elongated with an intramolecular overhang (see Figure 3a). Additionally, a quencher was introduced at the 5′‐ends of the strands. The intramolecular overhang allows the probe to form a stem‐loop structure, resulting in spatial proximity of the quencher to the fluorescent nucleoside. This design should enable efficient quenching of the fluorophore and result in minimized fluorescence emission from the free probes. Structurally similar oligonucleotides have been used in the past, for example, to detect maturation of pre‐miRNA.^[34]^ The five quencher probes (Figure 4) differ in the base sequence of the overhang. We calculated the secondary structures with UNAFold^[35]^ and selected them in such a way that only one secondary structure was found. Variation in the positioning of the quencher relative to the fluorophore by different RNA overhangs was necessary, since efficient energy transfer depends, among other things, on the spacing and spatial orientation of the chromophores relative to each other.^[36]^ Furthermore, it is important that the emission spectrum of the fluorophore and the absorption spectrum of the quencher have a sufficient overlap. We chose DABCYL as the quencher because it meets the necessary spectral characteristics and is commercially available as an NHS‐ester, making it easy to attach to RNA. The relevant spectra of the two chromophores are shown in Figure 3b. **Probes 3**–**7** (see Figure 4) were prepared by standard solid phase synthesis. For the attachment of the quencher, a C3 amino modifier was used, which was post‐synthetically labeled with a DABCYL‐NHS ester (for detailed information see Supporting Information). The short linker was intended to ensure a small distance between the fluorescent nucleobase and the quencher. However, we found in the NHS‐labeling reaction that the conventional protocols^[37]^ did not result in good yields. We believe that the reaction was inhibited due to the short linkers and high steric demand of the linked RNA, and therefore we had to adjust the reaction conditions. A significantly higher amount of organic solvent, a 60‐fold excess of NHS ester, and reaction times over several days were used. This allowed the labeling reaction to be completed in satisfactory yields of 30–40%.

After successful characterization of all five probes, fluorescence measurements were performed (Figure 4b). Our results show that for all probes the goal of reduced background fluorescence caused by the free RNA in solution was achieved. The fluorescence intensity increase – upon hybridization to **SRL abasic** – ranged from two‐fold (**probe 5**) to five‐fold (**probe 4**). At the same time, no increase in fluorescence was observed upon addition of **SRL**. It is notable that the **probes 3**, **5** and **6** which were predicted to have the most stable hairpin structures also resulted in the smallest fluorescence increase in our measurements. They are most likely too stable to interact with a complementary RNA. In contrast, **probes 4** and **7** showed the highest fluorescence after hybridization with **SRL abasic** and at the same time were predicted to form the least stable hairpins. However, the best results were obtained with **probe 4**. Although it did not show the highest absolute fluorescence after hybridization with **SRL abasic**, it showed the largest relative increase. A five‐fold increase was observed when **probe 4** was mixed with **SRL abasic**, whereas there was no increase after addition of **SRL**.

To our knowledge, this is the first time a probe is ever reported for indicating an abasic site that fluoresces exclusively when depurination has occurred and is also dark in the unhybridized state. In contrast to the previously reported probes, which also fluoresce unbound^[30]^ or lose fluorescence intensity after depurination,^[31]^ the probes developed here have clear advantages to enable selective visualization of abasic sites. In the next step, we tested whether the probes were suitable not only for visualizing the depurination of short RNA sequences but also the SRL full‐length sequence. Therefore, we synthesized **full‐length SRL** and **full‐length SRL abasic** (see Supporting Information). We performed hybridization experiments of **probes 3**, **4**, **5** and **7** with the long SRL complements. The experiments are shown in Figure S2 and demonstrate that our probes are also suitable for longer RNA sequences, as they show distinct differences in their fluorescence intensity in the different hybridization states, similar to the experiment shown in Figure 4.

**Figure 5:**
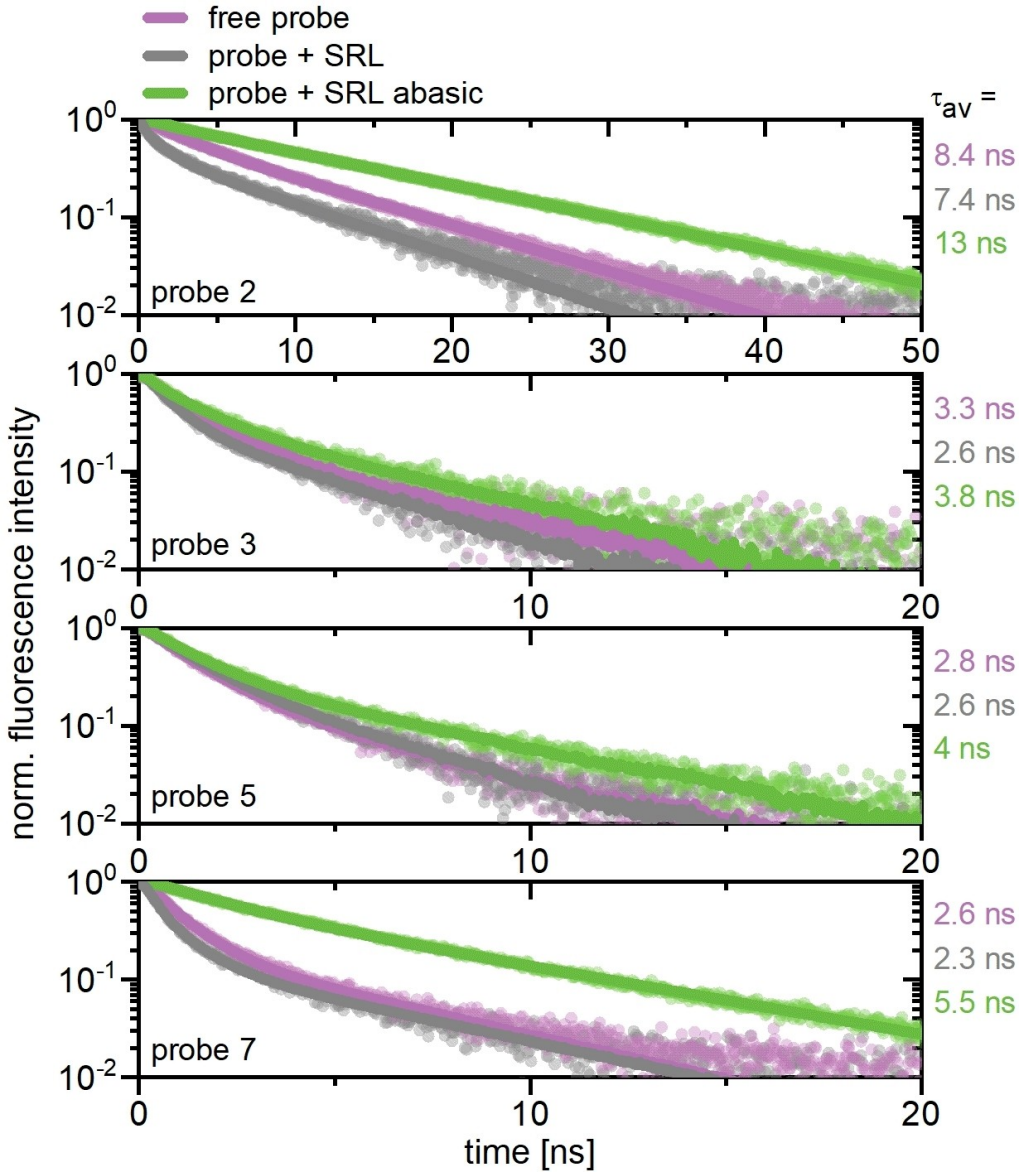
TCSPC measurements of the free and hybridized samples of the linear **probe 2**, and the stem‐loop **probes 3**, **5**, and **7**, with the DABCYL quencher attached. The obtained fits are represented by solid lines and the data by semi‐transparent points. The obtained intensity weighted average fluorescence lifetimes τ_av_ are provided to the right of the graphs.

In addition to steady state fluorescence measurements, we investigated whether the different probe designs also differed in their fluorescence lifetimes. For this purpose, we performed time‐correlated single photon counting (TCSPC) measurements with the linear **probes 1**–**2** and **probes 3**–**7** with the DABCYL quencher attached. The corresponding fluorescence decay curves of the free and hybridized states of **probe 2** and **probes 3**, **5**, and **7**, with different relative quencher positions, are shown in Figure 5 together with the obtained fits and determined average lifetimes. The remaining probe measurements can be found in the Supporting Information.

In either case, biexponential functions were applied to achieve adequate fit qualities. Hybridized to **SRL**, **probe 2** showed the fastest decay, which is in line with the lowest steady state emission detected. Although the free and the abasic matched state of **probe 2** were not distinguishable by their steady state emission, they differed in their fluorescence lifetimes of 7.4 ns and 8.4 ns, respectively. A differentiation is thus only possible by time‐resolved spectroscopic techniques.

The presence of the quencher in **probes 3**, **5**, and **7** can be directly recognized since the associated decay curves were all accelerated significantly, compared to **probe 2**. In any case, the probes hybridized to **SRL abasic** showed the slowest fluorescence decay. Interestingly, both the decays of the free probe states and those hybridized to **SRL** were much faster and quite similar, resembling the steady state observations. This suggests similarities in the quenching processes in the vicinity of either a matching base or DABCYL. The latter functions as energy acceptor that dissipates the energy to the surroundings as heat. In the free **probe 5**, direct electronic interactions between the label and the quencher enable non‐radiative energy transfer *via* contact quenching.^[38]^ Apparently, a similar energy transfer is achieved by base pairing with an adenine, since **probe 5** showed almost identical decays in its free state and hybridized to **SRL**. For **probes 3** and **7**, the quencher is not directly opposite of the label, resulting in a slightly longer average lifetime of the free state compared with the state hybridized with **SRL abasic**. If DABCYL is more distant to the label, it still acts as non‐radiative energy acceptor *via* Förster resonance energy transfer (FRET). Due to this proximity, also the decays of the probes hybridized to **SRL abasic** are accelerated.^[38]^ Overall, the TCSPC measurements show that the newly synthesized probes in this work are well‐suited to distinguish the depurinated SRL from both free probe and intact SRL. Thereby, both the pure fluorescence intensity and the fluorescence lifetime can be used as sensor readout.

**Figure 6:**
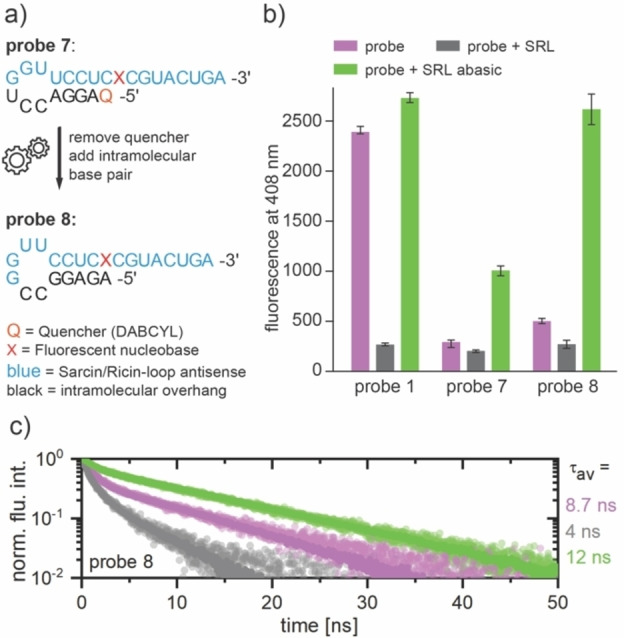
a) Design of new bright **probe 8** for optimized depurination detection. The former attached quencher is removed and replaced by an intramolecular base pair to keep the overall fluorescence high, while maintaining a low background signal. b) Comparison of fluorescence measurements of linear **probe 1**, quencher **probe 7** and bright **probe 8**. c_probe_=10 μM in CSH brain buffer (135 mM NaCl, 5.4 mM KCl, 1 mM MgCl_2_, 5 mM HEPES) at 37 °C, 2 eq counter strand RNA, λ_ex_=304 nm, v_ges_=100 μL. c) Fluorescence decay curves of the free and hybridized states of **probe 8** with determined average fluorescence lifetimes τ_av_.

Even though the probes synthesized so far already showed the expected behavior, we wanted to find a probe design to further improve the signal induction. Through our rational design, we managed to reduce the background fluorescence using a quencher, but at the same time, the maximum fluorescence hybridized with the **SRL** was decreased. The brightest synthesized quencher **probe 7** was less than half as bright as linear **probe 1** when **SRL abasic** was added. This observation is easily explained by the fact that although the distance between the fluorophore and the quencher increases after hybridization with a complementary strand, the two chromophores are not spaced far apart because they are attached to the same RNA strand. Consequently, we sought a solution to keep the background fluorescence low whilst increasing the maximum achievable signal to the original level after addition of **SRL abasic**. This led us to the very simple idea that the fluorescence of the synthesized nucleoside may be quenched not only by an organic quencher but also by base pairing. As we have shown several times in this study, the fluorescence of all probes is quenched by addition of a complementary base to the fluorescent nucleoside. Until now, however, a second strand has always been used for this purpose. Our goal was now to test whether fluorescence quenching can also be achieved by intramolecular base pairing. Our TCSPC measurements already showed that both the quencher and the base pairing must operate *via* a comparable mechanism (Figure 5). This gave us additional optimism that efficient quenching might also occur by an intramolecular overhang. Therefore, we removed the quencher in the next step and designed the intramolecular overhang allowing base pairing with the fluorescent nucleoside. (see Figure 6, left). After solid phase synthesis, we performed fluorescence measurements of the new **probe 8** under the same conditions as for the previous probes. The results are shown in Figure 6, right. Similar to the quencher probes, the free **probe 8** also showed a very low background signal in solution. We compared the newly synthesized **probe 8** with **probe 7**, which had the highest fluorescence signal after hybridization with the **SRL abasic** out of the quencher strands. Free **probe 7** had a background of 12% compared to linear **probe 1**. At the same time, the background fluorescence of **probe 8** was about 20%, which is of the same order of magnitude. All three compared strands have the lowest background fluorescence when hybridized with **SRL**. This further underscores their unique ability to distinguish depurinated from purinated RNAs. However, the biggest advantage of the new bright probe is its intensity after hybridization with **SRL abasic**. While **probe 7** only shows an intensity of 37% compared to linear **probe 1**, we were able to achieve 96% with **probe 8**, while at the same time the fluorescence of the free RNA strand remained at a very low level. Furthermore, the determined 12 ns fluorescence lifetime of **probe 8** hybridized with **SRL abasic** resembles the lifetimes of the probes without quencher (**probe 1**: 11.4 ns, **probe 2**: 13 ns) very well (Figure 6, bottom). In contrast to the probes with attached quencher, the free state of **probe 8** and the state hybridized to **SRL** with lifetimes of 8.8 ns and 4 ns, respectively, can be readily distinguished by time‐resolved spectroscopy. This shows that our carefully designed RNA architecture allowed us to selectively reduce the background fluorescence without affecting the signal enhancement indicating an abasic site. Therefore, the probes presented here are superior to any previously described probes which are intended for monitoring depurination.

## Conclusion

In this work, we describe the design and optimization of next generation RNA probes for monitoring depurination reactions on RNA strands. We used an SRL (sarcin/ricin loop) analog model system to demonstrate the unique fluorescent properties of the new probes. In contrast to previous methods to indicate depurination of SRL,[^30^, ^31^] we have developed for the first time an approach where a fluorescent signal is only displayed when depurination has really occurred. More precisely, our rational design allowed us to reduce the background fluorescence of the free RNA strand from 88% (probe 2 as literature known reference) to 20% (probe 8). Furthermore, we demonstrated that the different hybridization states can be distinguished not only by their fluorescence intensity but also by their fluorescence lifetimes using our new probes (see Figure 5). This is the first time a probe has been reported that fluoresces hybridized to an RNA containing an abasic site while having a low background fluorescence in the unbound state. For future experiments, we believe that the RNA probes presented here will find wide application in many biological investigations. Indeed, we anticipate that they could be used in vivo to monitor depurination at ribosomes in living cells following exposure to ricin‐like molecules.^[39]^

## Conflict of interest

The authors declare no conflict of interest.

1

## Supporting information

As a service to our authors and readers, this journal provides supporting information supplied by the authors. Such materials are peer reviewed and may be re‐organized for online delivery, but are not copy‐edited or typeset. Technical support issues arising from supporting information (other than missing files) should be addressed to the authors.

Supporting Information

## Data Availability

The data that support the findings of this study are available in the supplementary material of this article.

## References

[1] A. A. Szewczak , P. B. Moore , J. Mol. Biol. 1995, 247, 81–98. 7897662 10.1006/jmbi.1994.0124

[2] M. M. Yusupov , G. Z. Yusupova , A. Baucom , K. Lieberman , T. N. Earnest , J. H. D. Cate , H. F. Noller , Science. 2001, 292, 883–896. 11283358 10.1126/science.1060089

[3] A. A. Szewczak , P. B. Moore , Y. L. Chan , I. G. Wool , Proc. Natl. Acad. Sci. USA 1993, 90, 9581–9585. 8415744 10.1073/pnas.90.20.9581 PMC47613

[4] M. J. Walsh , J. E. Dodd , G. M. Hautbergue , Virulence 2013, 4, 774–784. 24071927 10.4161/viru.26399 PMC3925711

[5] Y. Endo , I. G. Wool , J. Biol. Chem. 1982, 257, 9054–9060. 7047533

[6] Y. Endo , K. Mitsui , M. Motizuki , K. Tsurugi , J. Biol. Chem. 1987, 262, 5908–5912. 3571242

[7] Y. Endo , K. Tsurugi , J. Biol. Chem. 1988, 263, 8735–8739. 3288622

[8] C. Brieke , F. Rohrbach , A. Gottschalk , G. Mayer , A. Heckel , Angew. Chem. Int. Ed. 2012, 51, 8446–8476; 10.1002/anie.201202134 22829531

[9] N. Ankenbruck , T. Courtney , Y. Naro , A. Deiters , Angew. Chem. Int. Ed. 2018, 57, 2768–2798; 10.1002/anie.201700171 PMC6026863 28521066

[10] F. Schäfer , J. Wagner , A. Knau , S. Dimmeler , A. Heckel , Angew. Chem. Int. Ed. 2013, 52, 13558–13561; 10.1002/anie.201307502 24174377

[11] K. Ballabh Joshi , A. Vlachos , V. Mikat , T. Deller , A. Heckel , Chem. Commun. 2012, 48, 2746–2748. 10.1039/c2cc16654b 22159276

[12] J. S. Rinne , T. P. Kaminski , U. Kubitscheck , A. Heckel , Chem. Commun. 2013, 49, 5375–5377. 10.1039/c3cc42420k 23652644

[13] P. G. Donlin-Asp , C. Polisseni , R. Klimek , A. Heckel , E. M. Schuman , Proc. Natl. Acad. Sci. USA 2021, 118, 2017578118. 10.1073/pnas.2017578118 PMC8020670 33771924

[14] R. Klimek , M. Wang , V. R. McKenney , E. M. Schuman , A. Heckel , Chem. Commun. 2021, 57, 615–618. 10.1039/d0cc06704k 33346255

[15] R. Klimek , P. G. Donlin-Asp , C. Polisseni , V. Hanff , E. M. Schuman , A. Heckel , Chem. Commun. 2021, 57, 12683–12686. 10.1039/d1cc05664f 34780585

[16] K. Rau , A. Rentmeister , ACS Cent. Sci. 2017, 3, 701–707. 28776011 10.1021/acscentsci.7b00251 PMC5532709

[17] J. T. George , S. G. Srivatsan , Chem. Commun. 2020, 56, 12307–12318. 10.1039/d0cc05228k PMC7611129 33026365

[18] J. Dietzsch , D. Bialas , J. Bandorf , F. Würthner , C. Höbartner , Angew. Chem. Int. Ed. 2022, 61, e202116783. 10.1002/anie.202116783 PMC9302137 34937127

[19] A. A. Tanpure , S. G. Srivatsan , ChemBioChem 2012, 13, 2392–2399. 23070860 10.1002/cbic.201200408

[20] A. Ovcharenko , F. P. Weissenboeck , A. Rentmeister , Angew. Chem. Int. Ed. 2021, 60, 4098–4103; 10.1002/anie.202013936 PMC7898847 33095964

[21] J. Mattay , M. Dittmar , A. Rentmeister , Curr. Opin. Chem. Biol. 2021, 63, 46–56. 33690011 10.1016/j.cbpa.2021.01.008

[22] J. T. George , S. G. Srivatsan , Chem. Commun. 2020, 56, 12319–12322. 10.1039/d0cc05092j PMC7611084 32939524

[23] H. Depmeier , E. Hoffmann , L. Bornewasser , S. Kath-Schorr , ChemBioChem 2021, 22, 2826–2847. 34043861 10.1002/cbic.202100161 PMC8518768

[24] C. Steinmetzger , I. Bessi , A. K. Lenz , C. Höbartner , Nucleic Acids Res. 2019, 47, 11538–11550. 31740962 10.1093/nar/gkz1084 PMC7145527

[25] C. Steinmetzger , N. Palanisamy , K. R. Gore , C. Höbartner , Chem. Eur. J. 2019, 25, 1931–1935. 30485561 10.1002/chem.201805882

[26] P. M. Sabale , U. B. Ambi , S. G. Srivatsan , ACS Omega 2018, 3, 15343–15352. 30556003 10.1021/acsomega.8b02550 PMC6289544

[27] A. Raulf , C. K. Spahn , P. J. M. Zessin , K. Finan , S. Bernhardt , A. Heckel , M. Heilemann , RSC Adv. 2014, 4, 30462–30466. 25580242 10.1039/c4ra01027b PMC4285124

[28] F. Eggert , K. Kulikov , C. Domnick , P. Leifels , S. Kath-Schorr , Methods 2017, 120, 17–27. 28454775 10.1016/j.ymeth.2017.04.021

[29] S. G. Srivatsan , H. Weizman , Y. Tor , Org. Biomol. Chem. 2008, 6, 1334–1338. 18385838 10.1039/b801054d PMC5263222

[30] S. G. Srivatsan , N. J. Greco , Y. Tor , Angew. Chem. Int. Ed. 2008, 47, 6661–6665; 10.1002/anie.200802199 PMC2633406 18683267

[31] A. A. Tanpure , P. Patheja , S. G. Srivatsan , Chem. Commun. 2012, 48, 501–503. 10.1039/c1cc16667k 22105782

[32] H. Vorbrüggen , K. Krolikiewicz , B. Bennua , Chem. Ber. 1981, 114, 1234–1255.

[33] H. Vorbrüggen , C. Ruh-Pohlenz , Org. React. 1999, 1–630.

[34] S. Sambandan , G. Akbalik , L. Kochen , J. Rinne , J. Kahlstatt , C. Glock , G. Tushev , B. Alvarez-Castelao , A. Heckel , E. M. Schuman , Science. 2017, 355, 634–637. 28183980 10.1126/science.aaf8995

[35] “UNAFold,” can be found under http://www.unafold.org/, **n.d**.

[36] I. Medintz , N. Hildebrandt , FRET – Förster Resonance Energy Transfer: From Theory to Applications, Wiley-VCH Verlag GmbH, Weinheim, Germany, 2013.

[37] Lumiprobe, “NHS ester labeling of amino biomolecules Biomolecule,” **2020**.

[38] R. M. Franzini , E. T. Kool , Bioconjugate Chem. 2011, 22, 1869–1877. 10.1021/bc2003567 PMC3178687 21870777

[39] M. Heumüller , C. Glock , V. Rangaraju , A. Biever , E. M. Schuman , Nat. Methods 2019, 16, 699–702. 31308551 10.1038/s41592-019-0468-x

