# Supplementary material for: RNA Probes for Visualization of Sarcin/ricin Loop Depurination without Background Fluorescence
Source: Chem Asian J. 2022 Nov 18;17(24):e202201077. doi: 10.1002/asia.202201077 (PMC10098603; doi:10.1002/asia.202201077)
Supplement: Supplementary file 1 — Supporting Information [file ASIA-17-0-s001.pdf]

CHEMISTRY
AN ASIAN JOURNAL

Supporting Information

RNA Probes for Visualization of Sarcin/ricin Loop Depurination without Background Fluorescence

Robin Klimek, Christoph Kaiser, Nina S. Murmann, Nina Kaltenschnee, Teresa Spanò, Josef Wachtveitl,* Erin M. Schuman,* and Alexander Heckel*This manuscript is part of an Indo-German special collection.© 2022 Wiley-VCH GmbH. This is an open access article under the terms of the Creative Commons Attribution License, which permits use, distribution and reproduction in any medium, provided the original work is properly cited.

Table of content

1. Chemical synthesis 2
2. Solid-phase synthesis: 6
3. Fluorescence measurements 8
4. Mass spectrometry and analytical HPLC 12
5. Mass spectra 14
6. References 20

1. Chemical synthesis

Compound **2**:

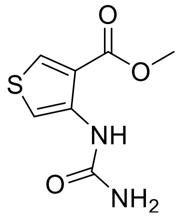

intermediate

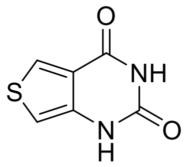

2

The synthesis was performed referring to a procedure known from literature.[1] To a solution of 4-aminothiophene-3 carboxylate hydrochloride **1** (2.97 g, 15.3 mmol, 1 eq) in aqueous acetid acid (50 mL, 50 wt%) was added KOCN (2.49 g, 15.3 mmol, 1 eq) dissolved in water (20 mL) over 10 minutes. The mixture was stirred at room temperature for 2 hours. Resulting solid was filtered and washed with cold water several times. The **intermediate** was dried in vacuo and isolated as brown solid.

Yield: 2.63 g (86%)

R_f (DCM/MeOH 95:5): 0.46

^{1}H-NMR (500 MHz, DMSO d-6): δ = 8.89 (s, 1H), 8.27 (d, J = 3.6 Hz, 1H), 7.65 (d, J = 6.0 Hz, 1H), 6.50 (s, 2H), 3.84 (s, 3H) ppm.

^{13}C- NMR (101 MHz, DMSO d-6): δ = 163.42, 155.52, 137.72, 133.06, 120.98, 106.65, 51.79 ppm.

MALDI-HRMS: m/z calculated for $C_7H_8N_2O_3S$ [M+H$^+$] = 201.03784, found [M+H$^+$] = 201.03283 Da (Δm = 0.00001, relative error 0.05 ppm).

The **intermediate** (950 mg, 4.7 mmol, 1 eq) was suspended in 40 ml dry methanol in a microwave vessel. NaOMe in MeOH (25 wt%, 5.43 mL, 23.7 mmol, 5 eq) was added and the mixture was refluxed for eight hours at 85 °C and afterwards stirred for 72 hours at room temperature. The resulting solid was filtered and washed with cold methanol several times. After drying in vacuo compound **2** was obtained as light brown solid.

Yield: 798 mg (quant.)

R_f (DCM/MeOH 95:5): 0.27

^{1}H-NMR (500MHz, D_2O): δ = 8.00 (d, J = 3.2 Hz, 1H), 6.72 (d, J = 3.2 Hz, 1H) ppm.

^{13}C-NMR (126 MHz, D_2O) δ = 168.41, 160.71, 142.47, 126.79, 122.49, 102.90 ppm.

ESI-MS: m/z calculated for $C_6H_4N_2O_2S$ [M+H$^+$] = 169.01, found [M+H$^+$] = 168.98.

MALDI-HRMS: m/z calculated for $C_6H_4N_2O_2S$ [M+H$^+$] = 169.00663, found [M+H$^+$] = 169.00697 (Δm = 0.00034 Da, relative error Δm/m = 2.01 ppm).

Compound **3**:

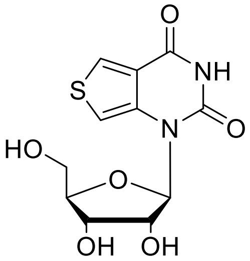

Fluorescent nucleobase **2** (2.00 g, 11.89 mmol, 1 eq) and (1-O-Acetyl-2,3,5-tri-O-benzoyl-*beta*-D-ribofuranose (9.00 g, 17.84 mmol, 1.5 eq) were suspended in 50 mL dry MeCN. Under continuous stirring bis(trimethylsilyl)acetamide (6.77 g, 33.3 mmol, 2.8 eq) was added and the mixture was heated to 100 °C under reflux for one hour. TMSOTf (3.44 g, 15.46 mmol, 1.3 eq) was added to the hot mixture, which caused it to clear up. After heating to 100 °C for another four hours the mixture was cooled to room temperature. It was diluted with dichloromethane und washed with saturated $NaHCO_3$ two times. The organic phase was dried over Na_2SO_4 and the solvent was removed under reduced pressure. The residue was redissolved in DCM and filtered over silica gel. The silica gel was washed using 5% MeOH in DCM until the eluent was not fluorescent anymore using 310 nm excitation. Removal of the solvent under reduced pressure led to the crude benzoyl protected intermediate (7.75 g).

The intermediate was dissolved in 100 mL MeOH. $MeNH_2$ in aqueous solution (40 wt%, 308.5 mmol) was added and the solution was stirred at room temperature overnight. The solvent was removed under reduced pressure and the crude product was purified using reversed phase flash column chromatography (water/MeOH). The solvent was removed under reduced pressure and **3** was obtained as white solid.

Yield: 1.45 g (42%)

R_f (DCM/MeOH 85:15): 0.15

^{1}H-NMR (400MHz, DMSO-d_6): δ = 11.29 (s, 1H), 8.43 (d, 1H, J = 3.1 Hz), 7.60 (d, 1H, J = 3.2 Hz), 6.07 (d, 1H, J = 7.0 Hz), 5.17 (d, 1H, J = 5.9 Hz), 5.10 (t, 1H, J = 4.9 Hz), 5.00 (d, 1H, J = 5.1 Hz), 4.46 (q, 1H, J = 6.4 Hz), 4.10-4.06 (m, 1H), 3.83-3.81 (m, 1H), 3.67-3.62 (m, 2H) ppm.

^{13}C-NMR (101 MHz) δ = 157.55, 150.58, 135.55, 130.06, 123.23, 106.52, 88.42, 84.95, 69.13, 68.11, 61.14 ppm.

MALDI-HRMS: m/z calculated for $C_{11}H_{12}N_2O_6S$ [M+Na$^+$] = 323.03083, found [M+Na$^+$] = 323.03074 (Δm = 0.00009 Da, relative error Δm/m = 0.28 ppm).

Compound **4**:

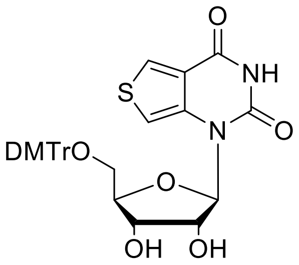

Synthesis was performed according to literature.[2] Compound **3** (121 mg, 403 µmol, 1 eq) was dissolved in 5 mL dry MeCN. Subsequently, DIPEA (260 mg, 2.01 mmol, 5 eq) and DMTr-Cl (143 mg, 423 µmol, 1.05 eq) were added and the mixture was stirred at room temperature for 24 hours. The reaction was quenched by adding 3 mL MeOH. The solvent was removed under reduced pressure and the crude product was purified via column chromatography (DCM/MeOH 95:5, 1% NEt_3). Nucleoside **4** was obtained as a white foam.

Yield: 126 mg (42%)

R_f (DCM/MeOH 95:5): 0.48

^{1}H-NMR (400MHz, DMSO-d_6): δ = 8.10 (d, 1H, J = 3.3 Hz), 7.50 (d, J = 3.3 Hz), 7.40-7.38 (m, 2H), 7.30-7.20 (m, 6H), 6.83-6.79 (m, 4H), 6.25 (d, 1H, J = 6.1 Hz), 4.79 (t, 1H, J = 6.5 Hz), 4.59 (dd, 1H, J = 6.6, 4.8 Hz), 4.10-4.07 (m, 1H), 3.77 (s, 6H), 3.54 (d, 2H, J = 2.7 Hz) ppm.

MALDI-HRMS: m/z calculated for $C_{32}H_{30}N_2O_8S$ [M+Na$^+$] = 625.16206, found 625.16120 (Δm = 0.00086 Da, relative error Δm/m = 0.07 ppm).

Compound **5**:

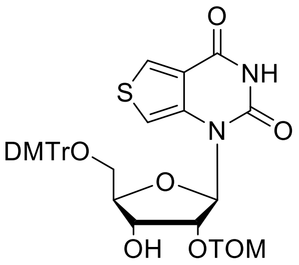

Synthesis was performed according to literature.[2] Nucleoside **4** (237 mg, 0.39 mmol, 1 eq) was dissolved in 5 mL anhydrous dichloroethane. To the stirring solution diisopropylethylamine (193 mg, 1.49 mmol, 3.8 eq) and dibutyltin dichloride (124 mg, 0.39 mmol, 1 eq) were added subsequently. After stirring for one hour at room temperature, the mixture was heated to 80 °C for 10 minutes and [(triisopropylsilyl)oxy]methyl chloride (114 mg, 0.51 mmol, 1.3 eq) was added to the hot solution. Heating was continued for another 20 minutes. After cooling to room temperature, it was diluted with dichloromethane and washed with $NaHCO_3$ (saturated). The aqueous phase was separated and extracted with dichloromethane two times. After the solvent was removed under reduced pressure the crude product was purified via column chromatography (cyclohexane/ethyl acetate, 1:1) to give **5** as a white foam.

Yield: 168 mg (52%)

R_f (CH/EtOAc 1:1): 0.42

^{1}H-NMR (400MHz, DMSO-d_6): δ = 8.41-8.39 (m, 1H), 7.39-7.35 (m, 3H), 7.29-7.22 (m, 8H), 6.87-6.84 (m, 4H), 6.21 (d, 1H, J = 6.6 Hz), 5.22 (d, 1H, J = 6.8 Hz), 4.95-4.91 (m, 2H), 4.83 (t, 1H, J = 6.7 Hz), 4.46-4.40 (m, 1H), 3.99-3.96 (m, 1H), 3.73 (s, 6H), 0.96-0.95 (m, 3H), 0.87-0.86 (m, 18H) ppm.

Compound **6**:

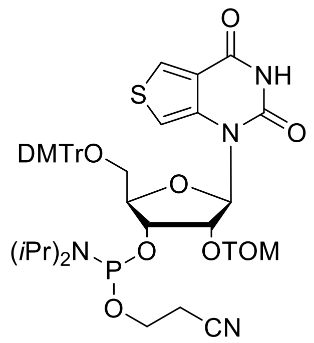

The synthesis was performed referring to a procedure known from literature.[2] TOM-protected nucleoside **5** (160 mg, 0.20 mmol, 1 eq) was dissolved in 5 mL dry dichloromethane. Diisopropylethylamine (96 mg, 0.41 mmol, 2 eq) and 2-cyanoethoxy-*N*,*N*-diisopropylaminochlorophosphine (131 mg, 1.01 mmol, 5 eq) were added subsequently under continuous stirring. The mixture was stirred at room temperature for 20 hours under an argon atmosphere. The solvent was removed under reduced pressure and the crude product was purified via column chromatography (cyclohexane/EtOAc, 2:1). The product was obtained as a white foam.

Yield: 174 mg (87%)

R_f (CH/EtOAc 1:1): 0.58

^{1}H-NMR (400MHz, DMSO-d_6): δ = 8.13 (t, 1H, J = 3.5 Hz), 7.52-7.49 (2d, 1H, J = 3.3 Hz), 7.42-7.38 (m, 2H), 7.32-7.23 (m, 7H), 6.84-6.79 (m, 4H), 6.40, (t, 1H, J = 6.8 Hz), 5.09 (t, 1H, J = 6.8 Hz), 4.99-4.91 (m, 2H), 4.77-4.67 (m, 1H), 4.26-4.21 (m, 1H), 3.99-3.83 (m, 2H), 3.79-3.78 (m, 6H), 2.67-2.64 (m, 1H), 2.29-2.25 (m, 1H), 1.21-1.15 (m, 8H), 1.02-1.01 (m, 8H), 0.93-0.90 (m, 21H) ppm.

^{31}P-NMR (120 MHz, $CDCl_3$): 150.88, 149.80 ppm.

MALDI-HRMS: m/z calculated for $C_{51}H_{69}N_4O_{10}PSSi$ [M+Na$^+$] = 1011.41335, found [M+Na$^+$] = 1011.41410 (Δm = 0.00075 Da, relative error Δm/m = 0.74 ppm).

2. Solid-phase synthesis:

Solid-phase synthesis was performed on an ABI392 instrument. Pac_2O (Merck) was used as capping reagent and 0.3 M BTT (*emp Biotech*) as activator. Coupling time for all amidites was 12 minutes. Synthesis was performed in DMTr-On mode. The cyanoethyl groups were removed with 20% diethylamine (*emp Biotech*) for 10 minutes. Cleavage from the solid phase was performed at 55 °C with aqueous ammonia (32%) (*Merck*) for 16 hours. After spin filtration, the solvent was removed at 4 °C using a vacuum concentrator (SpeedVac™, *Thermo Fischer*). Silyl protecting groups were cleaved using triethylamine (60 µl) and triethylaminohydrofluoride (75 µl) in DMSO (115 µl) for 150 minutes at 60 °C. To remove fluoride salts the oligonucleotides were dissolved in 0.3 M NaOAc (Merck) (25 µl). EtOH (*Sigma-Aldrich*), prechilled to -20 °C, 1 ml) was added. The mixture was cooled to -20 °C for at least 6 hours. The precipitant was pelletized by centrifugation at 4 °C, 20000 g for 20 minutes. The residue was redissolved in 0.3 M NaOAc and the precipitation steps were repeated 3 times. The oligonucleotides were purified on an Agilent 1200 equipped with a reversed phase *waters XBridge BEH* C18 OBD column (300 Å, 5 µm, 19x250 mm, 4 mL/min, 60 °C). As solvents 400 mM hexafluoroisopropanol (*Fluorochem*), 16.3 mM Et_3N (*Merck*), pH 8.3 and MeOH (*Fluka*) were used with a gradient from 5% to 100% MeOH in 22 minutes. The 5' DMTr-protecting groups were removed by incubating the oligonucleotides with 80% AcOH (1 ml) at room temperature for 20 minutes. The solvents were removed using a vacuum concentrator and the DMTr-off oligonucleotides were purified *via* reverse phase HPLC using the same conditions as described above.

Quencher labeling:

The oligonucleotide was dissolved in borate buffer (pH 8.45). A 60-fold excess of the Dabcyl-NHS (*Sigma-Aldrich*) in DMSO was added. The volume ratio of buffer to DMSO was 3:1. After incubation for approximately 16 hours at 35 °C, a 60-fold excess of the quencher in DMSO was added again and buffer was supplemented so that the 3:1 ratio was maintained. After incubating again for eight hours at 35 °C, additional quencher in DMSO and buffer was added a third time. After final incubation for another 16 hours at 35 °C the excess of quencher was removed by size exclusion chromatography via Sephadex™ G-25 M from *GE Healthcare*. The oligonucleotides were purified *via* reversed phase HPLC with a gradient from 5% to 50% MeOH in 13 minutes.

Table S1. Sequences and determined molecular masses of the synthesized oligonucleotide **probes 1-8** and hybridization strands SRL and SRL abasic.

	**mass calc. [M-H]⁻**	**mass found [M-H]⁻**
**probe 1**<br>5‘- GGU UCC UCX CGU ACU GA -3‘	3722.4	3723.9
**probe 2**<br>5‘- UCC UCX CGU ACU -3’	5392.7	5394.3
**probe 3**<br>5‘- QAG AGG CCG GUU CCU CXC GUA CUG A -3’	8101.1	8102.1
**probe 4**<br>5‘- QGA GGC AGU GGU UCC UCX CGU ACU GA -3‘	8447.2	8448.2
**probe 5**<br>5‘- QGA GGC CGG UUC CUC XCG UAC UGA -3‘	7772.1	7772.0
**probe 6**<br>5’- QAG GAU GGU UCC UCX CGU ACU GA -3’	7452.0	7451.9
**probe 7**<br>5’- QAG GAC CUG GUU CCU CXC GUA CUG A -3’	8062.1	8063.1
**probe 8**<br>5’- AGA GGC CGG UUC CUC XCG UAC UGA -3’	7850.0	7851.0
**SRL**<br>5’- AGU ACG AGA GGA -3’	3919.6	3920.1
**SRL abasic**<br>5’- AGU ACG YGA GGA -3’	3770.6	3771.1
**full-length SRL**<br>5‘- CUG CUC AGU ACG AGA GGA ACC GCA G - 3‘	8055.2	8056.2
**full-length SRL abasic**<br>5‘- CUG CUC AGU ACG YGA GGA ACC GCA G - 3‘	7907.1	7907.3

A = rA (Bz) CE-Phosphoramidite (*Biosearch Technologies*)

C = rC (Ac) CE-Phosphoramidite (*Biosearch Technologies*)

G = rG (dmf) CE-Phosphoramidite (*Biosearch Technologies*)

U = U CE-Phosphoramidite (*Biosearch Technologies*)

X = Compound (**6**)

Q = Fmoc-Amino-DMT C-3 CED phosphoramidite (*ChemGenes*) labeled with DABCYL NHS (*Sigma Aldrich*)

Y = dSpacer CE Phosphoramidite (*GLEN Research*)

3. Fluorescence measurements

For steady state fluorescence intensity measurements the final concentration of the probes was 10 µM (n = 1 nmol) and for the counterstrands SRL and SRL abasic 20 µM (n = 2 nmol). The final volume used was 100 µl and the solutions were prepared in CSH Brain Buffer, having a final salt concentration of 135 mM NaCl, 5.4 mM KCl, 1 mM $MgCl_2$, and 5 mM HEPES at pH 7.4. Measurements were performed on a *Tecan infinite M200PRO* plate reader at 37 °C. 304 nm was used for excitation and fluorescence intensity at 408 nm was used for evaluation. Every measurement was repeated 3-5 times.

The time-correlated single photon counting (TCSPC) experiments were conducted with an FT100 spectrometer (PicoQuant, Berlin). For excitation, a pulsed LED PLS310 with a central wavelength of 310 nm and a pulse duration of 800 ps was applied, controlled by a PDL800-D driver (PicoQuant, Berlin). Time-resolved fluorescence measurements were acquired with software TimeHarp260 (PicoQuant, Berlin). The instrument response function (IRF) was measured by the scattered light of TiO_2 dispersed in ethanol. For the sample fluorescence measurements, a UVB390 filter was used to cut off the excitation stray light and the excitation. The concentrations of the labeled probes were 2 µM and the counter strands were provided in excess (3 µM). All of the samples were prepared in CSH brain buffer in 4x10 mm quartz glass cuvettes. Exponential fitting of the data was performed with the software FluoFit 4.6 (PicoQuant, Berlin).[3]

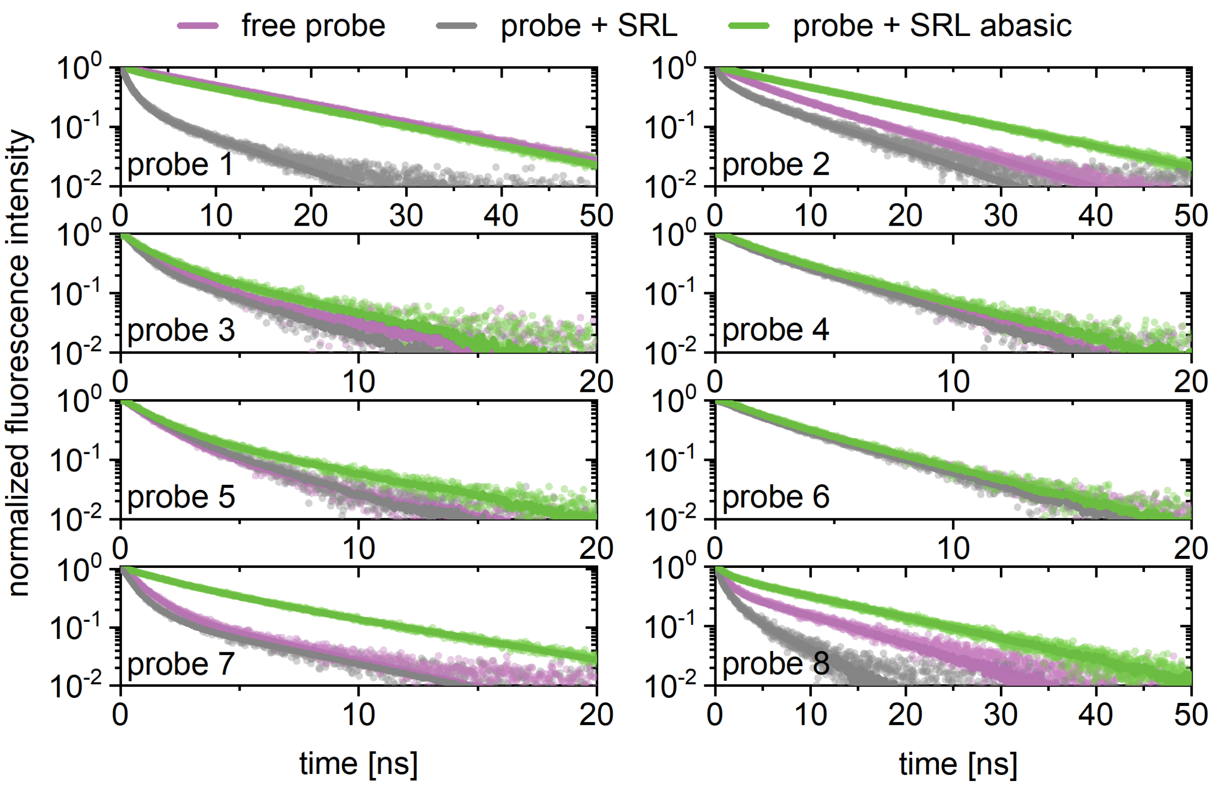

Figure S1. Fluorescence decay curves of the **free probes** (magenta), the hybridized states with **SRL** (gray) and with **SRL abasic** (green) of the **probes 1-8**. The semi-transparent dots represent the data points and the solid line the obtained multiexponential fits.

Table S2. Fluorescence decay fit parameters for all studied **probes 1-8** and their hybridization states. t_i represent the lifetime components obtained from biexponential fitting provided with their relative amplitudes A_i, t_{av} is the average fluorescence lifetime and c^2 the goodness of fit criterion.

**Probe**		**τ_1 [ns] (A_1 [%])**	**τ_2 [ns] / A_2 [%]**	**τ_{av}**	**χ^2**
**1**	free	14 (99)	1.8 (1)	14	1.05
	+ SRL	8 (69)	1.2 (31)	6	1.16
	+SRL abasic	13.8 (97)	1.5 (3)	13.5	1.05
**2**	free	10.2 (69)	4.4 (31)	8.4	1.01
	+ SRL	9 (75)	2.6 (25)	7.4	1.00
	+SRL abasic	13.2 (98)	3.3 (2)	13	1.01
**3**	free	6.1 (51)	1.4 (49)	3.8	1.05
	+ SRL	3.5 (68)	0.6 (32)	2.6	1.13
	+SRL abasic	4.8 (71)	1.2 (29)	3.8	1.10

**4**	free	3.8 (86)	0.9 (14)	3.4	1.01
	+ SRL	3.5 (57)	0.7 (43)	3.2	1.01
	+SRL abasic	4.4 (79)	1.4 (21)	3.8	1.14
**5**	free	4.3 (52)	1.2 (48)	2.8	1.02
	+ SRL	3.7 (59)	1.1 (41)	2.6	1.11
	+SRL abasic	5.4 (66)	1.2 (34)	4	1.10
**6**	free	5.1 (52)	2.2 (48)	3.7	1.06
	+ SRL	4.2 (67)	1.5 (33)	3.4	1.02
	+SRL abasic	2.5 (57)	5.5 (43)	3.8	1.04
**7**	free	1.1 (64)	5.3 (36)	2.6	1.08
	+ SRL	0.8 (63)	4.9 (37)	2.3	1.03
	+SRL abasic	7.2 (59)	3.1 (41)	5.5	1.04
**8**	free	10 (86)	1 (14)	8.7	1.11
	+ SRL	5.4 (69)	1.1 (31)	4	1.04
		12.5 (95)	1.1 (5)	12	1.07

Fluorescence measurements with **full-length SRL** and **full-length SRL abasic**:

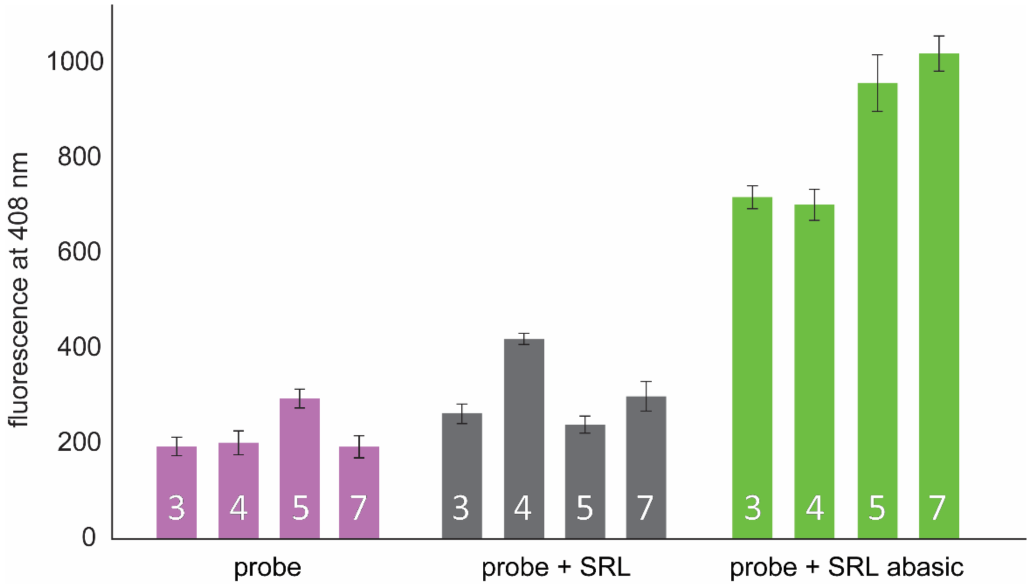

Figure S2. Probes 3, 4, 5 and 7 and their fluorescence properties when hybridized to complementary full-length SRL and full-length SRL abasic RNA. c_{probe} = 10 µM in CSH brain buffer (135 mM NaCl, 5.4 mM KCl, 1 mM $MgCl_2$, 5 mM HEPES) at 37 °C, 2 eq. counter strand RNA, λ_{ex} = 304 nm, v_{ges} = 100 µL.

Fluorescence measurements of **probe 6** with different mixing ratios of **SRL** and **SRL abasic**:

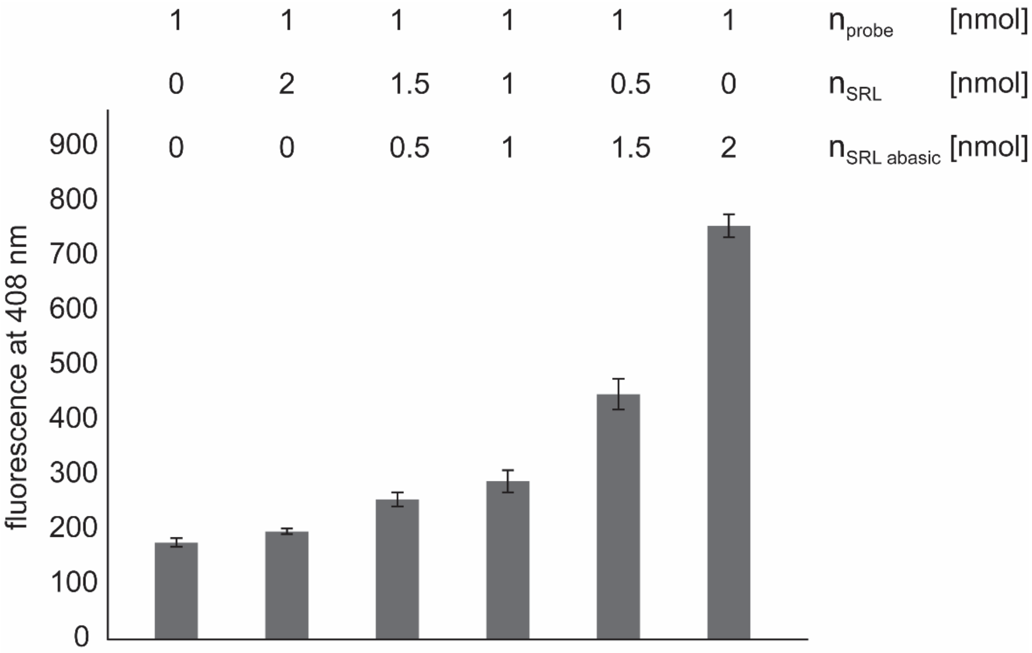

Figure S3. Probe 6 and its fluorescence properties when hybridized to different mixing ratios of complementary **SRL** and **SRL abasic** RNA. c_{probe} = 10 μM in CSH brain buffer (135 mM NaCl, 5.4 mM KCl, 1 mM $MgCl_2$, 5 mM HEPES) at 37 °C, λ_{ex} = 304 nm, v_{ges} = 100 μL.

4. Mass spectrometry and analytical HPLC

All mass spectra were measured on a Bruker *micrOTOF-Q in* negative ionization mode. Electrospray ionization (ESI) was used for ion generation. Analytical RP-HPLC was performed on an Agilent 1200 equipped with a BEH C18 OBD (300 Å, 3.5 µm, 4.6x250 mm, 1 mL/min, 60 °C). As solvents 400 mM hexafluoroisopropanol (fluorochem), 16.3 mM Et_3N (Merck), pH 8.3 and MeOH (Fluka) were used with a gradient from 5% to 100% MeOH in 39 minutes.

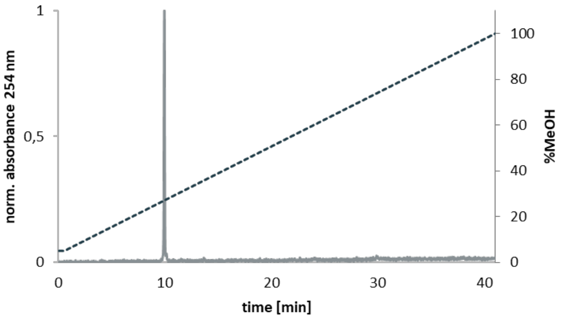

Figure S4. Analytical HPLC of **SRL**.

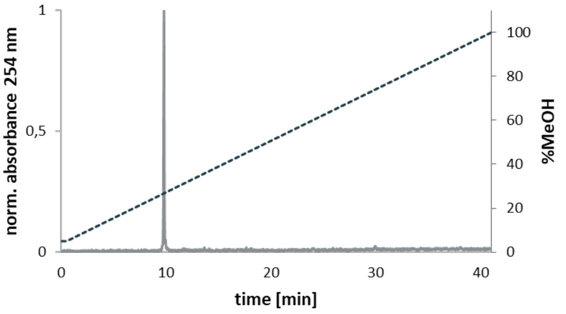

Figure S5. Analytical HPLC of **SRL abasic**.

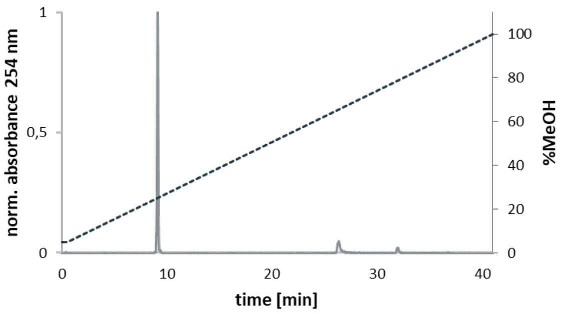

Figure S6. Analytical HPLC of **probe 1**.

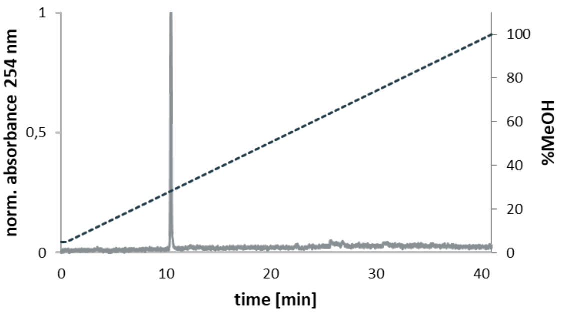

Figure S7. Analytical HPLC of **probe 2**.

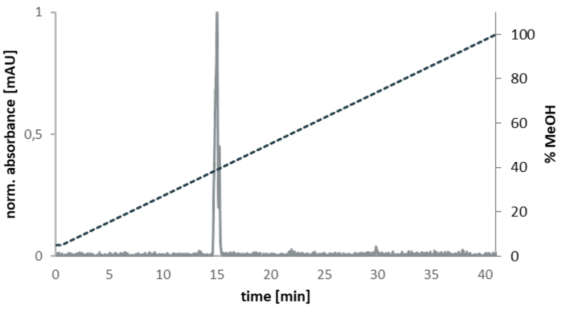

Figure S8. Analytical HPLC of **probe 3**.

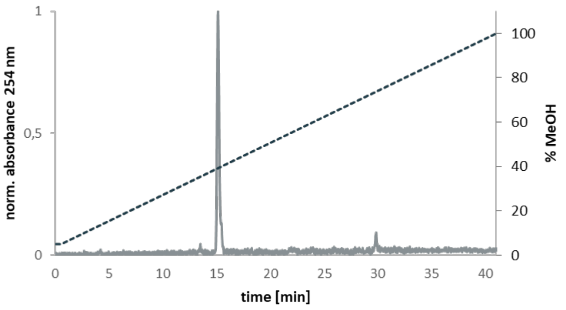

Figure S9. Analytical HPLC of **probe 4**.

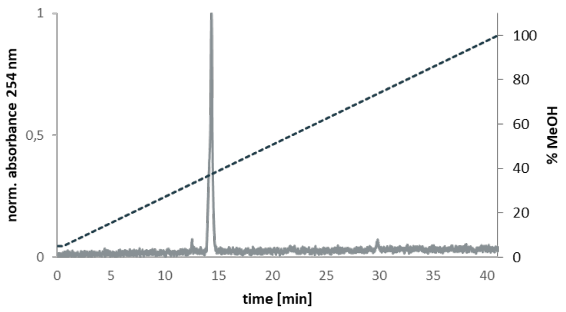

Figure S10. Analytical HPLC of **probe 5**.

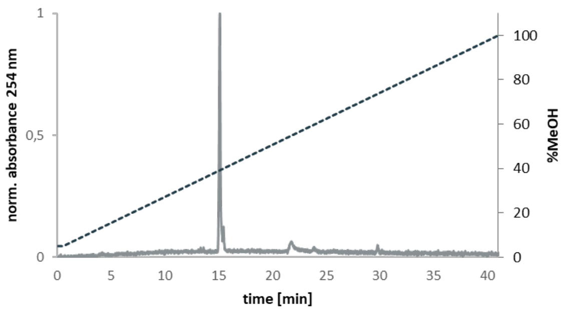

Figure S11. Analytical HPLC of **probe 6**.

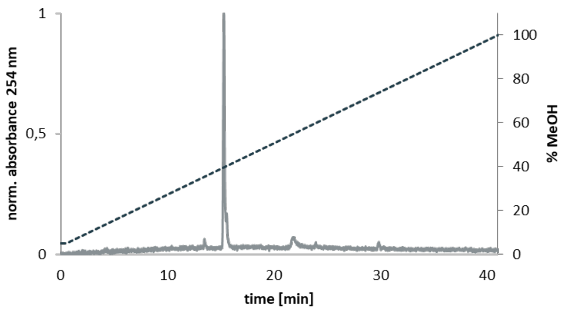

Figure S11. Analytical HPLC of **probe 7**.

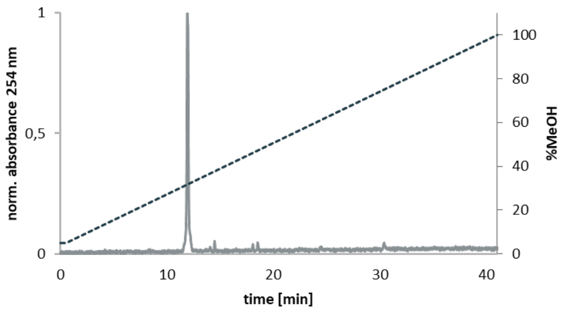

Figure S12. Analytical HPLC of **probe 8**.

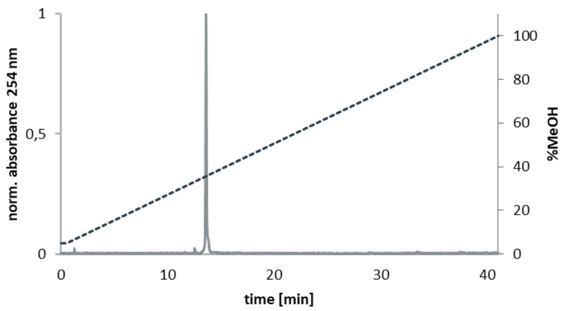

Figure S13. Analytical HPLC of **full-length SRL**.

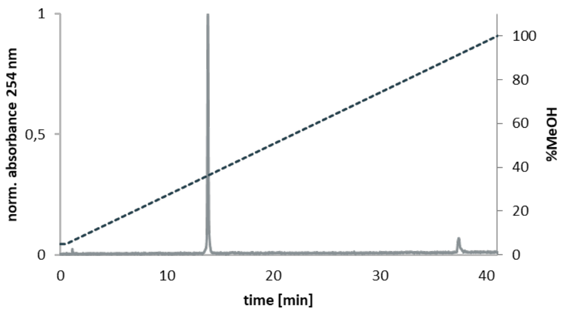

Figure S14. Analytical HPLC of **full-length SRL abasic**.

5. Mass spectra

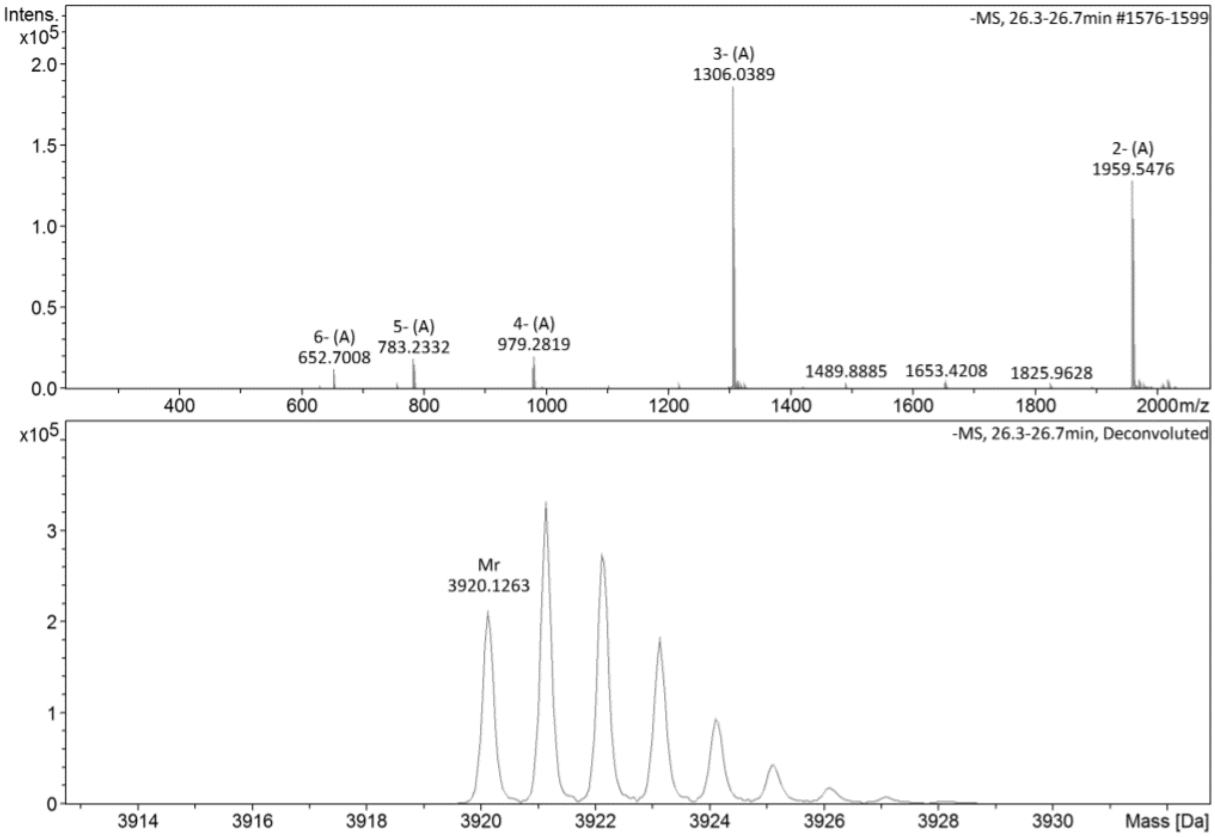

Figure S15. ESI-MS spectrum of **SRL**. Expected mass: [M-H]$^-$ = 3919.3 Da.

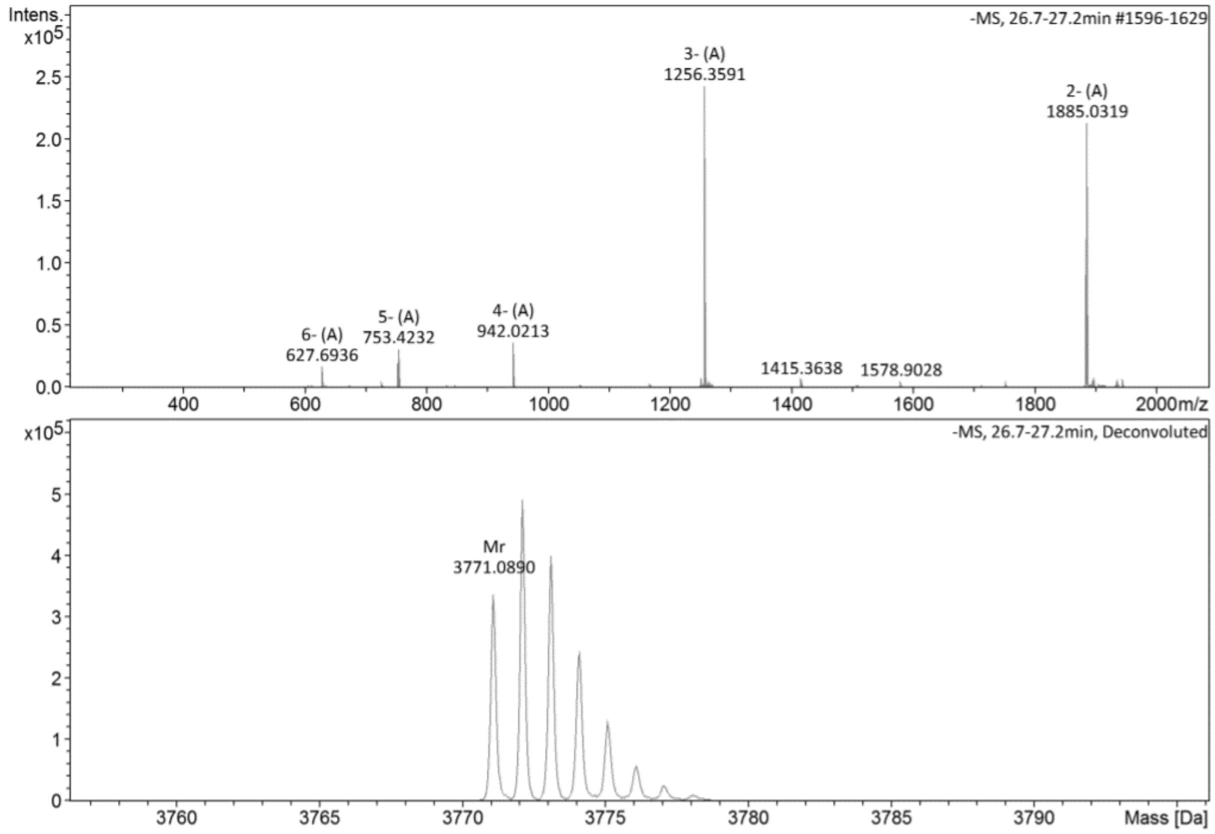

Figure S16. ESI-MS spectrum of **SRL abasic**. Expected mass: [M-H]$^-$ = 3772.4 Da.

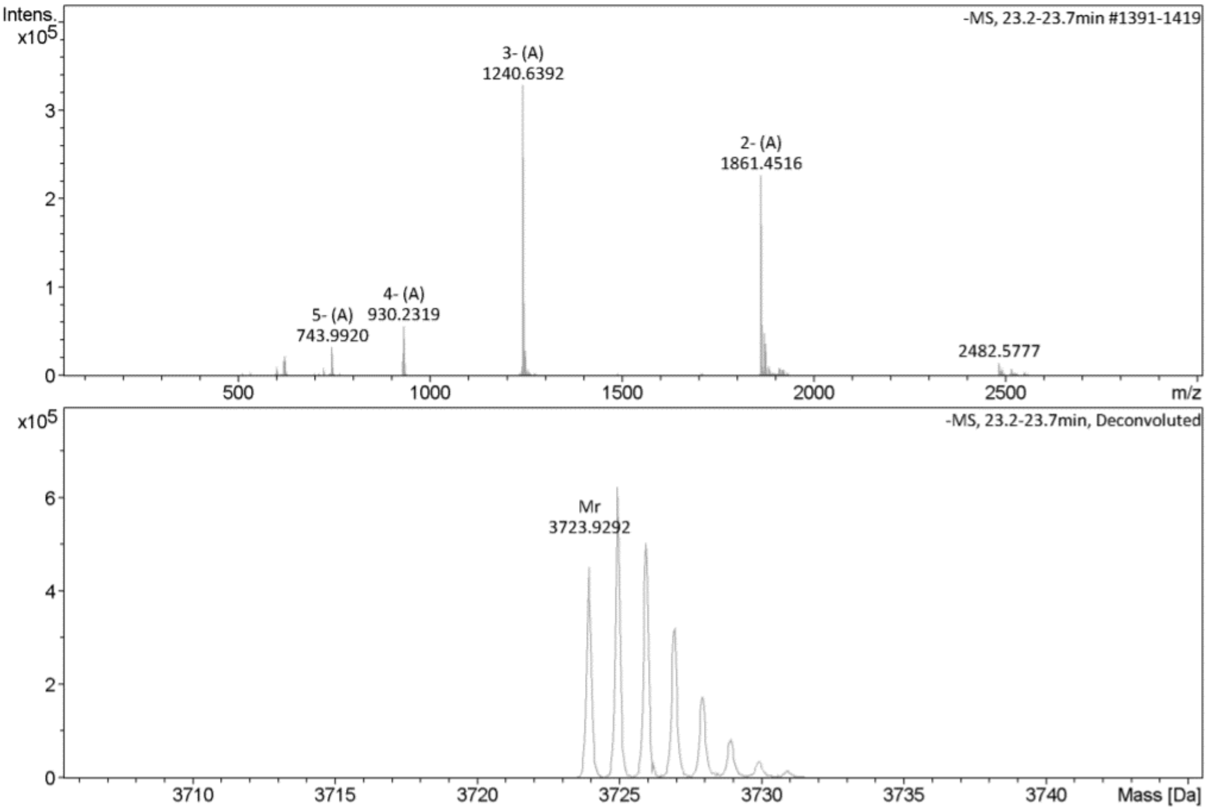

Figure S17. ESI-MS spectrum of **probe 1**. Expected mass: $[M-H]^-$ = 3722.4 Da.

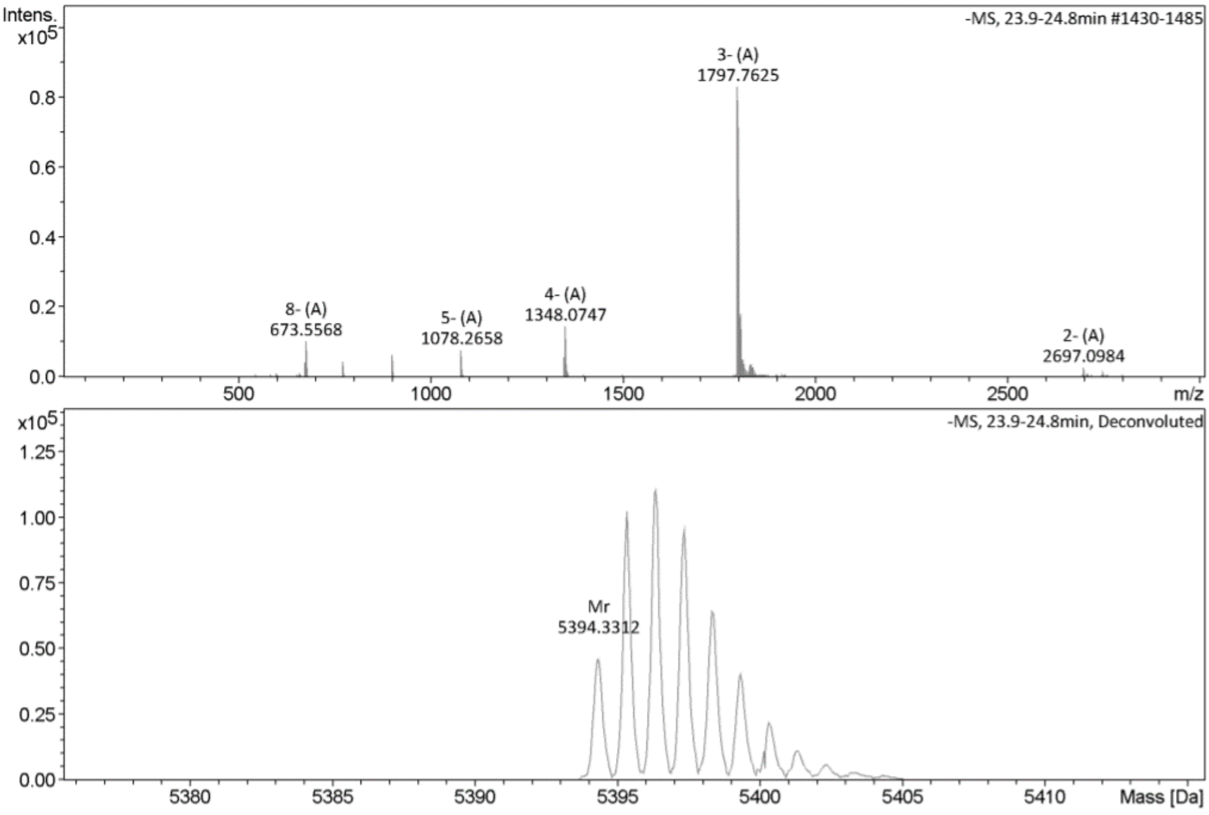

Figure S18. ESI-MS spectrum of **probe 2**. Expected mass: $[M-H]^-$ = 5392.7 Da.

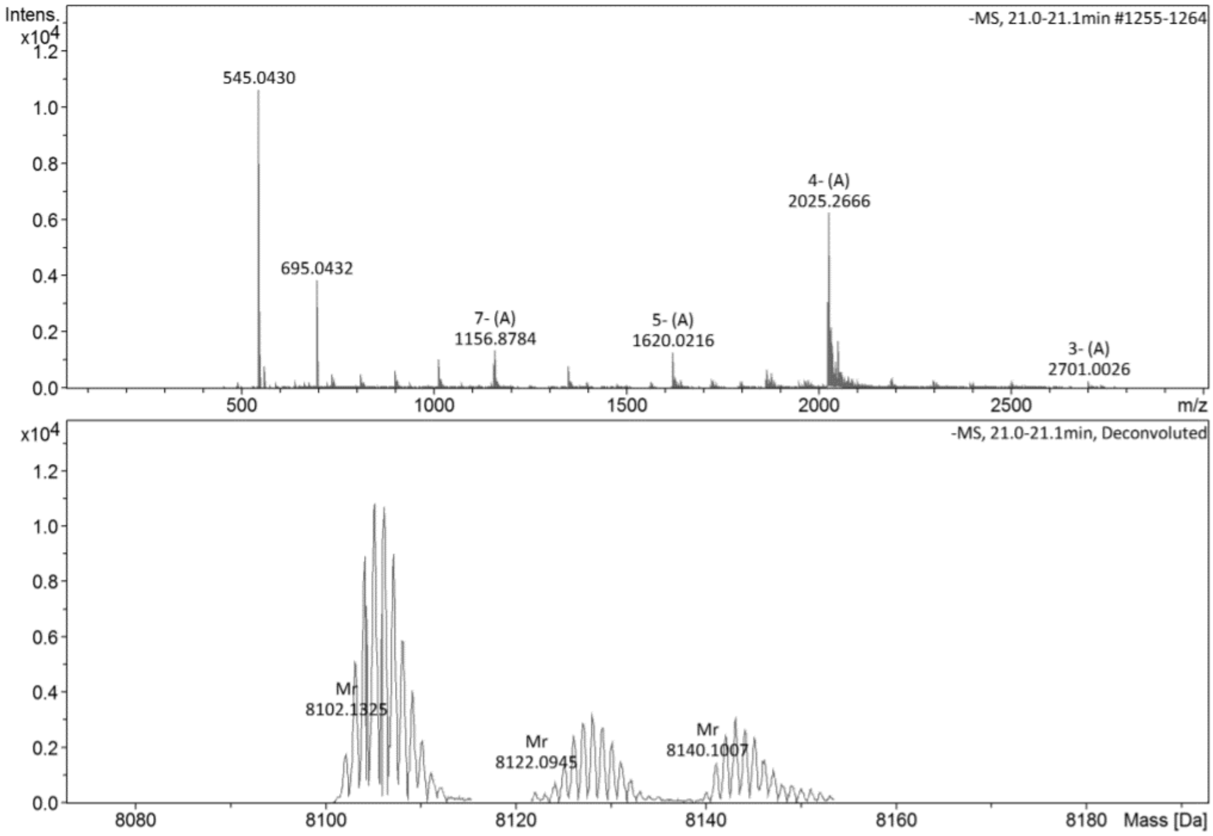

Figure S19. ESI-MS spectrum of **probe 3**. Expected mass: $[M\text{-}H]^-$ = 8101.1 Da.

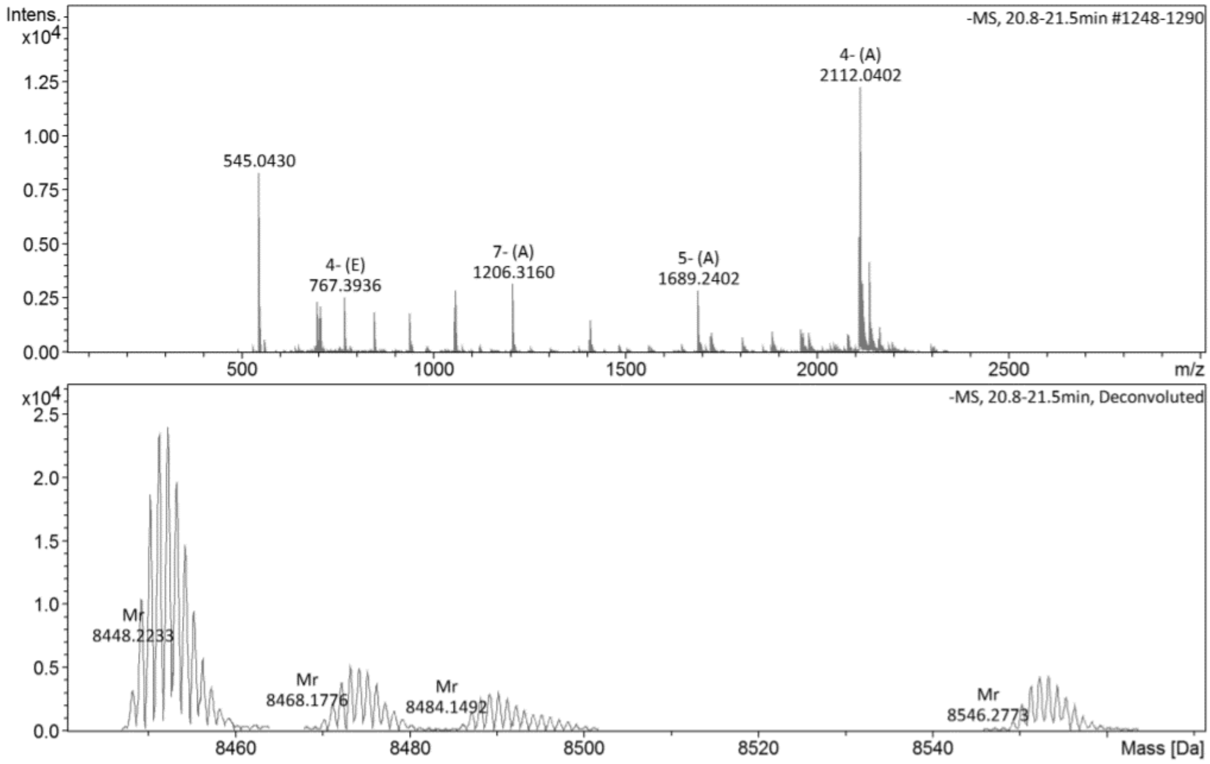

Figure S20. ESI-MS spectrum of **probe 4**. Expected mass: $[M\text{-}H]^-$ = 8447.2 Da.

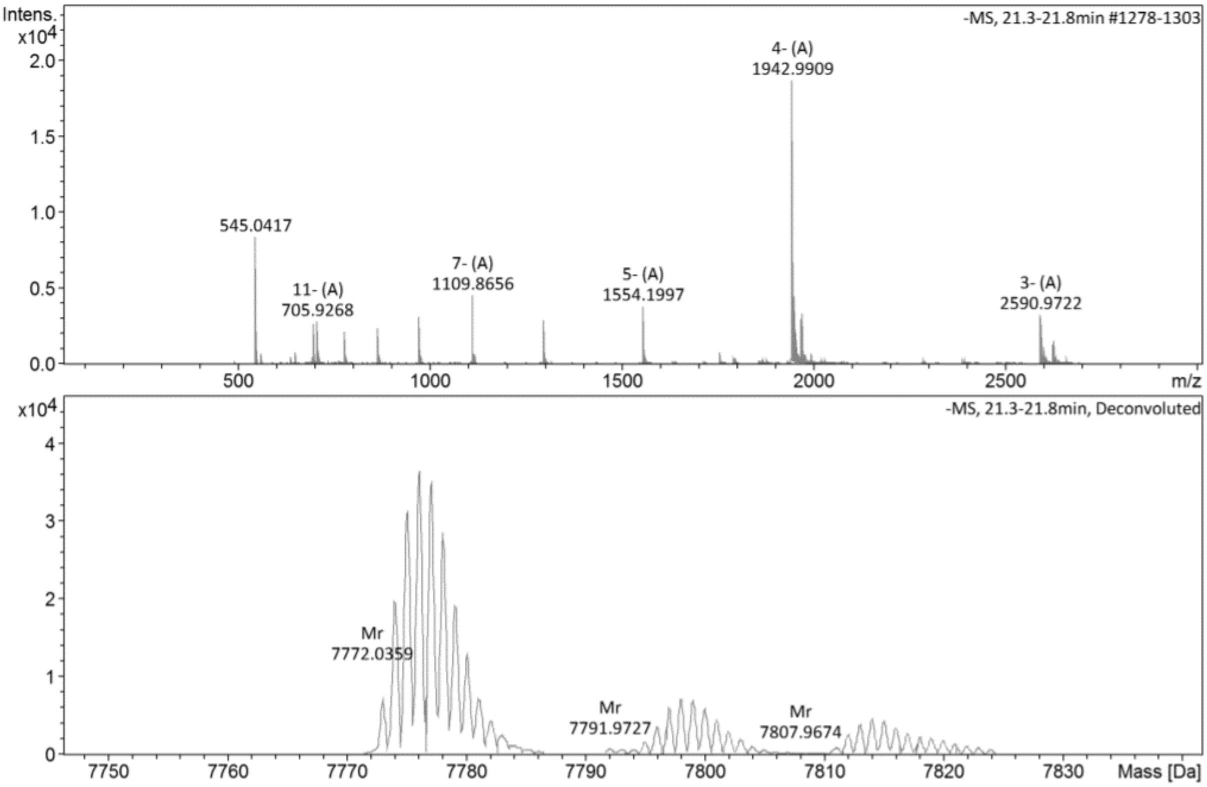

Figure S21. ESI-MS spectrum of **probe 5**. Expected mass: [M-H]$^-$ = 7772.1 Da.

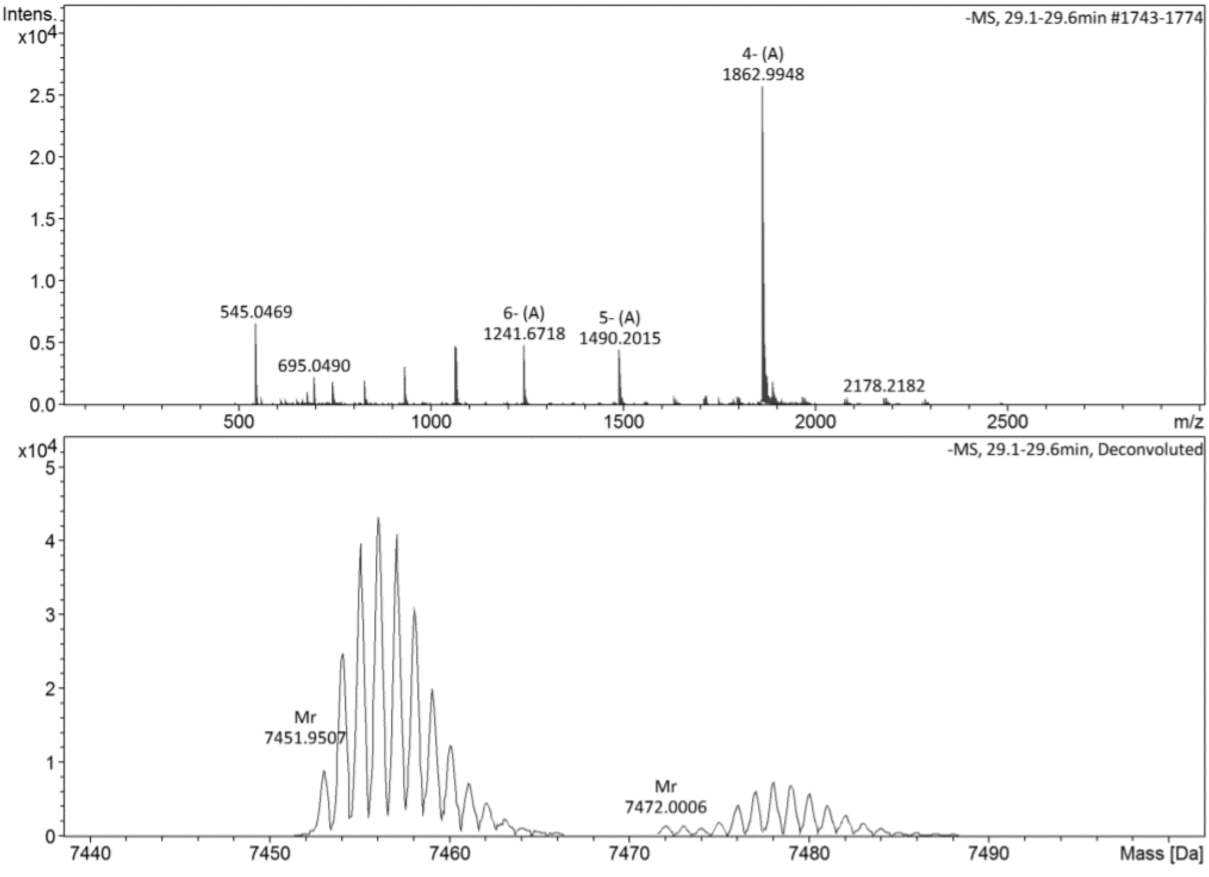

Figure S22. ESI-MS spectrum of **probe 6**. Expected mass: [M-H]$^-$ = 7452.0 Da.

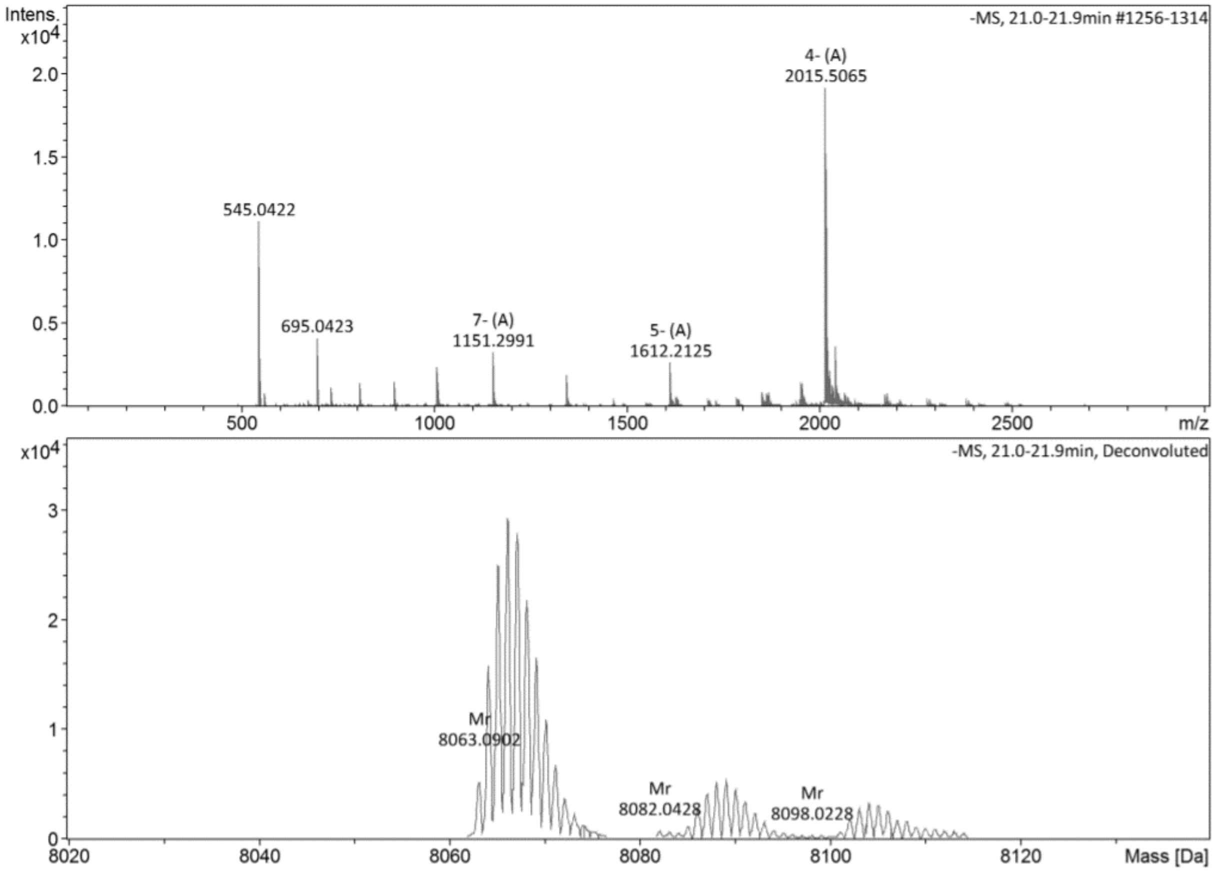

Figure S23. ESI-MS spectrum of **probe 7**. Expected mass: $[M-H]^-$ = 8062.1 Da.

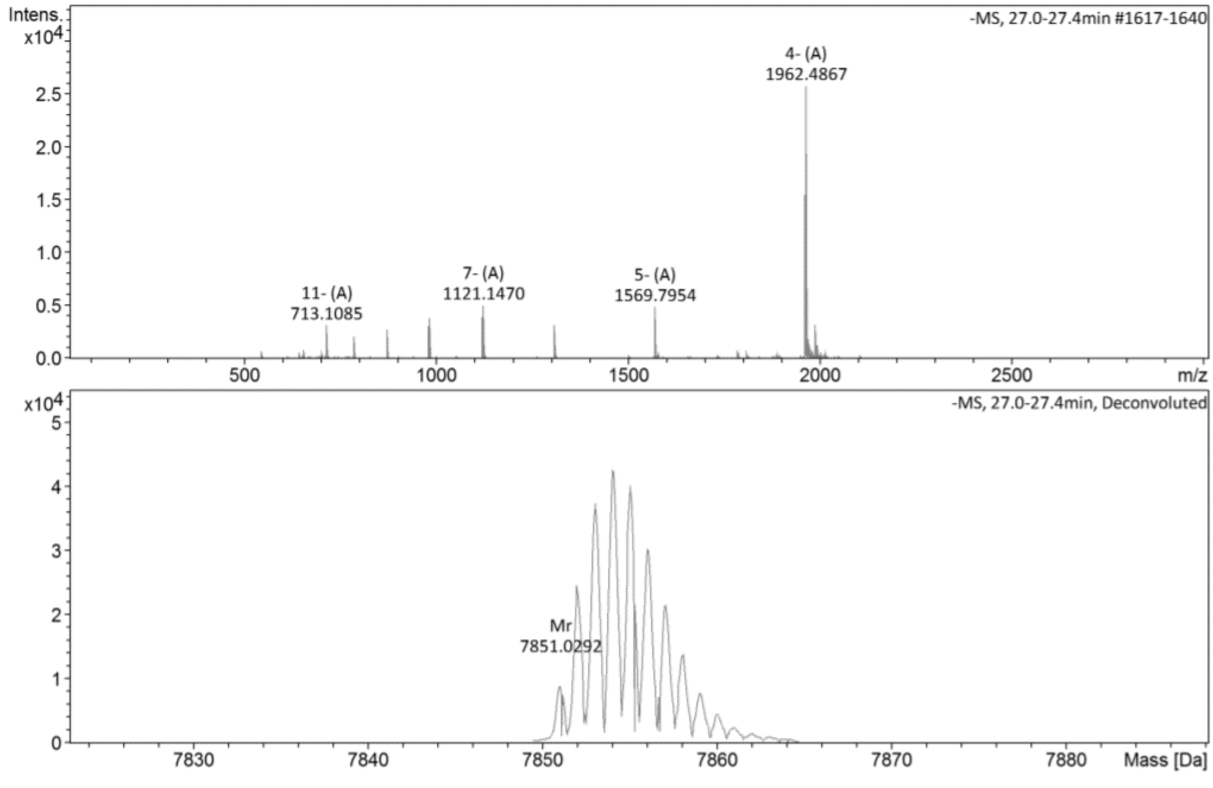

Figure S24. ESI-MS spectrum of **probe 8**. Expected mass: $[M-H]^-$ = 7850.0 Da.

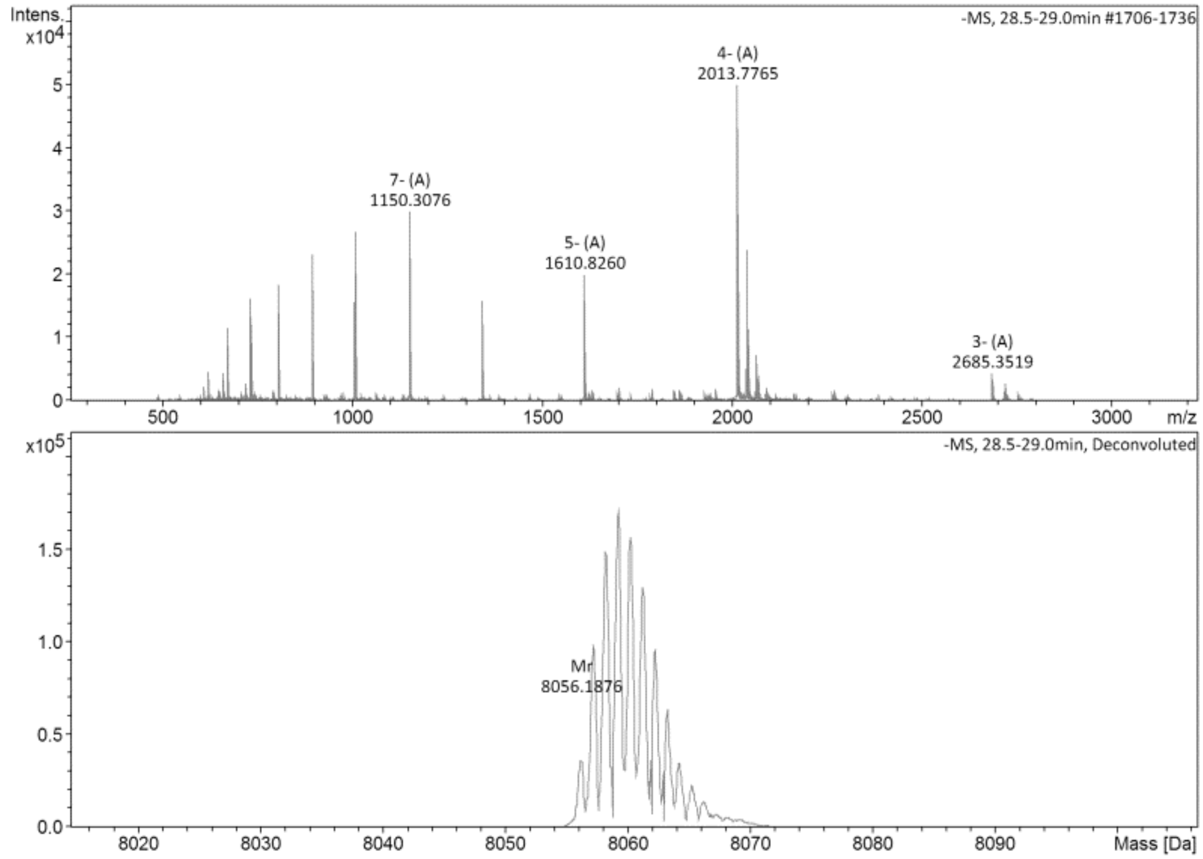

Figure S25. ESI-MS spectrum of **full-length SRL**. Expected mass: $[M\text{-}H]^-$ = 8055.2 Da.

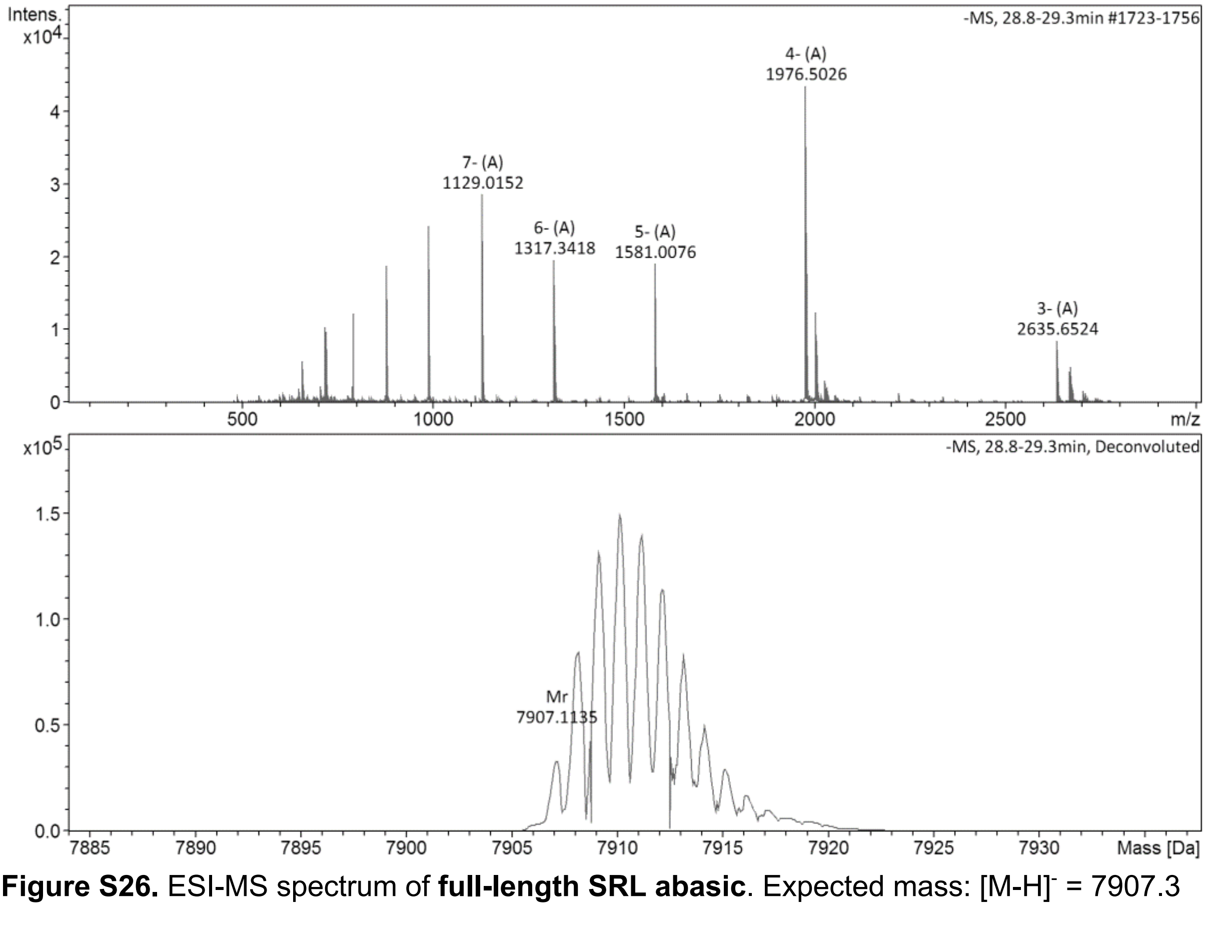

Figure S26. ESI-MS spectrum of **full-length SRL abasic**. Expected mass: $[M\text{-}H]^-$ = 7907.3

6. References

[1] S. G. Srivatsan, H. Weizman, Y. Tor, *Org. Biomol. Chem.* **2008**, *6*, 1334–1338.

[2] S. G. Srivatsan, N. J. Greco, Y. Tor, *Angew. Chem. Int. Ed.* **2008**, *47*, 6661–6665.

[3] J. Enderlein, R. Erdmann, *Opt. Commun.* **1997**, *134*, 371–378.
